# Structure-Guided Synthesis of FK506 and FK520 Analogs with Increased Selectivity Exhibit *In Vivo* Therapeutic Efficacy against Cryptococcus

**DOI:** 10.1128/mbio.01049-22

**Published:** 2022-05-23

**Authors:** Michael J. Hoy, Eunchong Park, Hyunji Lee, Won Young Lim, D. Christopher Cole, Nicholas D. DeBouver, Benjamin G. Bobay, Phillip G. Pierce, David Fox, Maria Ciofani, Praveen R. Juvvadi, William Steinbach, Jiyong Hong, Joseph Heitman

**Affiliations:** a Department of Molecular Genetics and Microbiology, Duke University Medical Center, Durham, North Carolina, USA; b Department of Immunology, Duke University Medical Center, Durham, North Carolina, USA; c Department of Chemistry, Duke University, Durham, North Carolina, USA; d Division of Pediatric Infectious Diseases, Department of Pediatrics, Duke University Medical Center, Durham, North Carolina, USA; e UCB Biosciences, Bainbridge Island, Washington, USA; f Seattle Structural Genomics Center for Infectious Disease (SSGCID), Seattle, Washington, USA; g Duke University NMR Center, Durham, North Carolina, USA; University of British Columbia

**Keywords:** X-ray crystallography, antifungal, antifungal resistance, antifungal susceptibility testing, ascomycin, calcineurin, tacrolimus

## Abstract

Calcineurin is an essential virulence factor that is conserved across human fungal pathogens, including Cryptococcus neoformans, Aspergillus fumigatus, and Candida albicans. Although an excellent target for antifungal drug development, the serine-threonine phosphatase activity of calcineurin is conserved in mammals, and inhibition of this activity results in immunosuppression. FK506 (tacrolimus) is a naturally produced macrocyclic compound that inhibits calcineurin by binding to the immunophilin FKBP12. Previously, our fungal calcineurin-FK506-FKBP12 structure-based approaches identified a nonconserved region of FKBP12 that can be exploited for fungus-specific targeting. These studies led to the design of an FK506 analog, APX879, modified at the C-22 position, which was less immunosuppressive yet maintained antifungal activity. We now report high-resolution protein crystal structures of fungal FKBP12 and a human truncated calcineurin-FKBP12 bound to a natural FK506 analog, FK520 (ascomycin). Based on information from these structures and the success of APX879, we synthesized and screened a novel panel of C-22-modified compounds derived from both FK506 and FK520. One compound, JH-FK-05, demonstrates broad-spectrum antifungal activity *in vitro* and is nonimmunosuppressive *in vivo*. In murine models of pulmonary and disseminated C. neoformans infection, JH-FK-05 treatment significantly reduced fungal burden and extended animal survival alone and in combination with fluconazole. Furthermore, molecular dynamic simulations performed with JH-FK-05 binding to fungal and human FKBP12 identified additional residues outside the C-22 and C-21 positions that could be modified to generate novel FK506 analogs with improved antifungal activity.

## INTRODUCTION

Invasive fungal infections represent a major global health burden. Many of the deadliest fungal infections, such as cryptococcal meningoencephalitis, occur in an increasing immunocompromised patient population receiving organ transplants, suffering from HIV/AIDS, treated with corticosteroids, or undergoing cancer chemotherapy. In fact, Cryptococcus neoformans infection is one of the leading causes of death in HIV/AIDS patients and is most common in areas where HIV/AIDS is widespread (1). Mortality rates as high as 70% with C. neoformans and another ubiquitous fungal pathogen, Candida albicans, present a complicated treatment challenge (2). In many cases, physicians must treat these infections with combination therapies of antifungal drugs due to the lack of fungus-specific drug targets and the associated difficulty in developing antifungal drugs with low toxicity. A rise in drug resistance across pathogenic fungi such as Candida sp. and Aspergillus fumigatus further exacerbates this issue (3). Within the space of FDA-approved antifungal drugs, clinicians have very limited options. Only 4 main classes of antifungal drugs (polyenes, azoles, pyrimidine analogs, and echinocandins) are approved for treatment of systemic fungal infection, and each treatment suffers from issues related to high toxicity, bioavailability, or innate and acquired resistance in many species (4). There is an urgent need to develop new antifungal strategies and identify novel targets to address the increasing global threat of deadly invasive fungal diseases.

Calcineurin signaling in C. neoformans, A. fumigatus, and C. albicans, as well as in other fungal pathogens, is highly conserved and leads to the activation of virulence genes and proteins that are essential for the organism’s growth at host body temperature, hyphal development, and survival in serum, respectively (5–9). These essential roles in key virulence attributes make calcineurin an excellent target for antifungal drug development (10, 11). Calcineurin is a serine-threonine specific protein phosphatase that is a heterodimer consisting of a catalytic subunit, CnA, and a regulatory subunit, CnB, and is activated in the presence of calcium and calmodulin (12). When activated in many pathogenic fungi, calcineurin dephosphorylates the transcription factor Crz1 and triggers its nuclear translocation and transcription of downstream target genes (13–15). FK506 (tacrolimus) is a naturally occurring calcineurin inhibitor that is a product of several Streptomyces species and has potent antifungal activity (8, 16, 17). FK506 acts on fungal cells by binding to FK506 binding protein 12 (FKBP12) and forming a complex that binds to and inhibits calcineurin by sterically blocking substrate access to the active site (18, 19). FKBP12 is a member of a larger family of proteins called immunophilins that bind to immunosuppressive molecules and mediate their activity (20).

Calcineurin signaling is conserved in humans and mediates critical pathways involved in growth and proliferation. Additionally, the mechanism of calcineurin inhibition is also conserved via FK506-FKBP12 complex binding to calcineurin (19). In humans, calcineurin is critical for T-cell activation and the initiation of immune responses (21). Activated calcineurin dephosphorylates the Crz1 homolog NFAT, which promotes expression of immune response genes, such as that encoding the cytokine interleukin 2 (IL-2) (22, 23). As a result, the potent immunosuppressive activity of FK506 in humans makes it unsuitable to treat patients with fungal infections (24). In fact, due to this robust immunosuppressive activity, FK506 is FDA approved as a posttransplant drug and is administered to patients to prevent host rejection of a variety of transplanted organs and tissues (25, 26).

FK506 is not the only naturally occurring calcineurin inhibitor and FKBP12-binding molecule. FK520 (ascomycin) is a natural FK506 analog that is modified at the C-21 position with an ethyl group replacing the C-21 allyl group in FK506 (27). This C-21 modification of FK506 results in a molecule with similarly potent antifungal activity and modestly reduced immunosuppressive activity (28). In this case, modification of the C-21 position introduced a slight but detectable fungal specificity. Although the antifungal and immunosuppressive activity of FK520 has been well-characterized, few studies have focused on developing fungus-specific variants of FK520 (29). The C-21 position of FK520 is on the calcineurin-facing region of the molecule and may influence formation of the ternary inhibitory complex. Conformationally, modifications made to the C-22 position of FK506 and FK520 could be influenced by the differences at the C-21 residue.

Previous studies have sought to develop nonimmunosuppressive calcineurin inhibitors for the treatment of fungal infections. Minor modifications to the structure of FK506/FK520 have been shown to significantly alter both its immunosuppressive and antifungal activity. L-685,818 is a nonimmunosuppressive analog of FK520 that was shown to maintain antifungal activity via calcineurin inhibition despite having no activity against human calcineurin (17, 30). Structurally quite similar to FK520, L-685,818 differs only in being hydroxylated at the C-18 position. L-685,818 binds human FKBP12 identically to FK506, and the C-18 and C-21 modifications are both exposed on the calcineurin-interacting effector surface (31, 32). While the precise molecular mechanism by which L-685,818 disrupts ternary complex formation is still unknown and complex chemical synthesis has limited further study, it has been hypothesized the introduction of the polar hydroxyl group may disrupt a hydrophobic interface that would otherwise dock with calcineurin.

We previously demonstrated that APX879, a C-22 acetylhydrazone-modified FK506 analog, exhibits a clear increase in fungal specificity that translates into therapeutic efficacy (33). Structures of fungal FKBP12-FK506 and ternary structures of calcineurin-FK506-FKBP12 from A. fumigatus, C. neoformans, Coccidioides immitis, and C. albicans led to the structure-guided design of APX879 (33, 34). Key structural differences in the 80s loop of FKBP12 proximal to the FK506-binding pocket led to fungus-specific differences in the FKBP12-FK506-calcineurin interface. Specifically, fungal Phe88 or mammalian His88 residues of FKBP12 introduced differences in distance to the C-22 acetylhydrazone of APX879 and presented a clear opportunity to exploit this for increasing fungal specificity. Moreover, protein-inhibitor complex crystal structures and molecular dynamic (MD) simulations identified multiple residues around the molecule that could enhance fungal specificity (35). Compared to FK506, APX879 exhibits an ~70-fold reduction in immunosuppression, corresponding to an overall 4-fold increase in fungal specificity determined by its therapeutic index score (33). Guided by the success of C-22 modification in the previous study, we generated a series of second-generation FK506 analogs, as well as the first examples of C-22-modified FK520 analogs. Here, we report the first crystal structure of a fungal FKBP12 bound to FK520 that was instrumental in the design of FK520-based analogs. Strikingly, one such FK520 analog described here is nonimmunosuppressive *in vivo* and efficacious in multiple models of C. neoformans infection. Our current advances in structural work and medicinal chemistry provide further evidence that iterative development of FK506/FK520 analogs can increase fungal specificity and drive these compounds into a therapeutic window that can be utilized to treat invasive fungal infections.

## RESULTS

### A. fumigatus FKBP12-bound FK520 X-ray crystal structure reveals conserved binding pocket structure.

**FIG 1:**
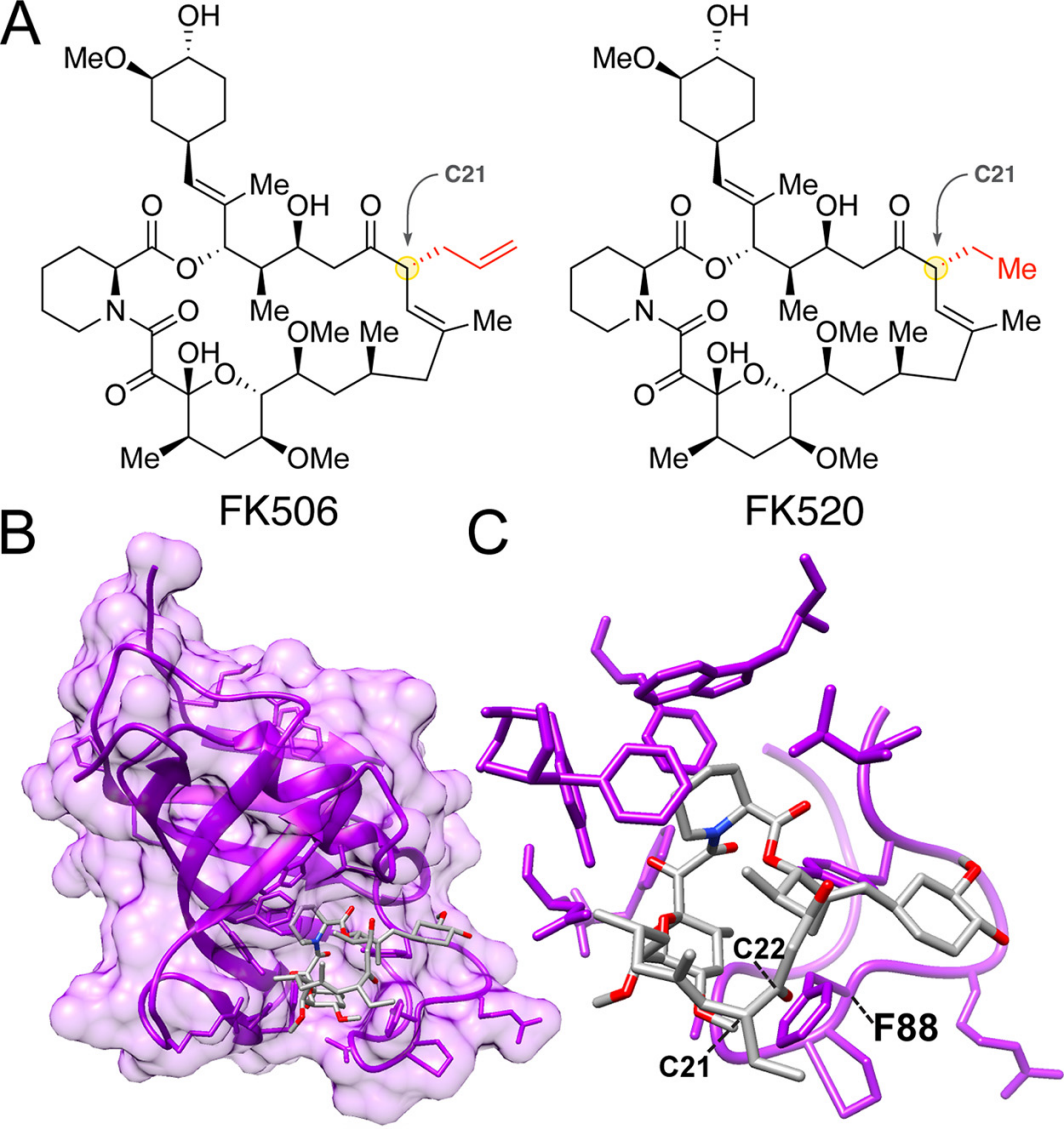
Crystal structure of Aspergillus fumigatus FKBP12 bound to FK520. (A) Chemical structures of FK506 (tacrolimus) and FK520 (ascomycin). Structural differences at the C-21 residue are highlighted in red. (B) Protein X-ray crystal structure of A. fumigatus FKBP12 (purple) bound to FK520 (gray) characterized at a resolution of 1.7 Å. (C) Higher-resolution focus of FK520 bound to the FKBP12 ligand-binding pocket. Both C-22 and C-21 of FK520 approach key fungus-specific residue F88 in the 80s loop of FKBP12.

We previously solved X-ray crystal structures of FKBP12-FK506-calcineurin ternary complexes from divergent human-pathogenic fungi (33). FK506 and FK520 are structurally similar but differ at the C-21 position, where FK520 lacks the terminal alkene of FK506 (Fig. 1A). To gain better insight into the interactions between FK520 and fungal FKBP12, we first determined the X-ray crystal structure at 1.7 Å of A. fumigatus FKBP12 bound to FK520 (PDB identifier [ID] 7U0S). A. fumigatus was selected for FKBP12 structure analysis due to the readiness with which protein crystals form and the presence of key conserved fungal residues in the 80s loop. The FKBP12-binding motif of FK506 and FK520 is conserved and thus FK520 binds identically to A. fumigatus FKBP12 (Fig. 1B and C). We have previously shown that the 80s loop of FKBP12 in fungi is a critical region for facilitating the formation of FKBP12-inhibitor-calcineurin complex (33). Phe88 is a conserved residue in many pathogenic fungi that differs from the mammalian His88 residue. Both C-21 and C-22 of FK520/FK506 converge on this differential residue within 5 to 8 Å and represent excellent targets for synthetic modification.

Due to inherent challenges in forming the full-length ternary complex for crystallography, we next generated a novel “mini-calcineurin” as an alternative approach to capture FK520 bound to human FKBP12 and calcineurin (see Fig. S1 in the supplemental material). The mini-calcineurin construct consists of the full-length CnB subunit fused C-terminally to the calcineurin B binding helix (BBH; A. fumigatus residues 335 to 370, Homo sapiens residues 337 to 372) of CnA, lacking the CnA catalytic domain present in previous structures. Constructs for both human and A. fumigatus were generated and high-resolution ternary structures of each protein bound to FK520 (PDB ID 7U0T) and FK506 (PDB ID 7U0U), respectively, were characterized (Table S1). Remarkably, overlays with bovine FKBP12-FK506-calcineurin (PDB ID 1TCO) reveal consistent conformation of the mini-calcineurin structure, demonstrating that the key molecular interactions between FKBP12-FK520 and calcineurin are largely attributable to the hydrophobic interface presented by CnB bound to the extended CnA alpha-helical arm. The root mean square deviation (RMSD) value for the overlay of the A. fumigatus mini-calcineurin was 0.90 Å, consistent with previously reported values of structural similarity (33). The human mini-calcineurin RMSD value was 0.57 Å, indicative of a high degree of structural similarity and that the truncation of CnA did not significantly alter the overall binding structure of the complex. Each of these novel FK520-bound structures represents a key addition to the tools necessary for structure-guided design of both FK506/FK520 analogs that show increased fungal selectivity.

10.1128/mbio.01049-22.1 FIG S1 Mini-calcineurin crystal structures of human and Aspergillus fumigatus protein bound to FK520 and FK506. Protein crystal structures for each organism presented with overlay of bovine full-length calcineurin ternary complex structure CN-FKBP12-FK506 (PDB ID 1TCO) in gray. (A) Structure of human mini-calcineurin complex (PDB ID 7U0T) consisting of CnB (red) and CnA B binding helix (BBH; pink), FKBP12 (blue), and FK520 (green). (B) Structure of A. fumigatus mini-calcineurin complex (PDB ID 7U0U) consisting of CnB (red) and CnA B binding helix (BBH; pink), FKBP12 (blue), and FK506 (green). (C) Protein expression construct of full-length calcineurin subunits A and B and mini-calcineurin containing CnB and CnA BBH linked by 17 amino acids. Download FIG S1, PDF file, 0.8 MB. Copyright © 2022 Hoy et al. 2022 Hoy et al. https://creativecommons.org/licenses/by/4.0/ This content is distributed under the terms of the Creative Commons Attribution 4.0 International license.

10.1128/mbio.01049-22.5 TABLE S1 Crystallographic data and refinement statistics. Download Table S1, DOCX file, 0.02 MB. Copyright © 2022 Hoy et al. 2022 Hoy et al. https://creativecommons.org/licenses/by/4.0/ This content is distributed under the terms of the Creative Commons Attribution 4.0 International license.

### Chemical synthesis of novel FK506 and FK520 analogs modified at the C-22 position.

**FIG 2:**
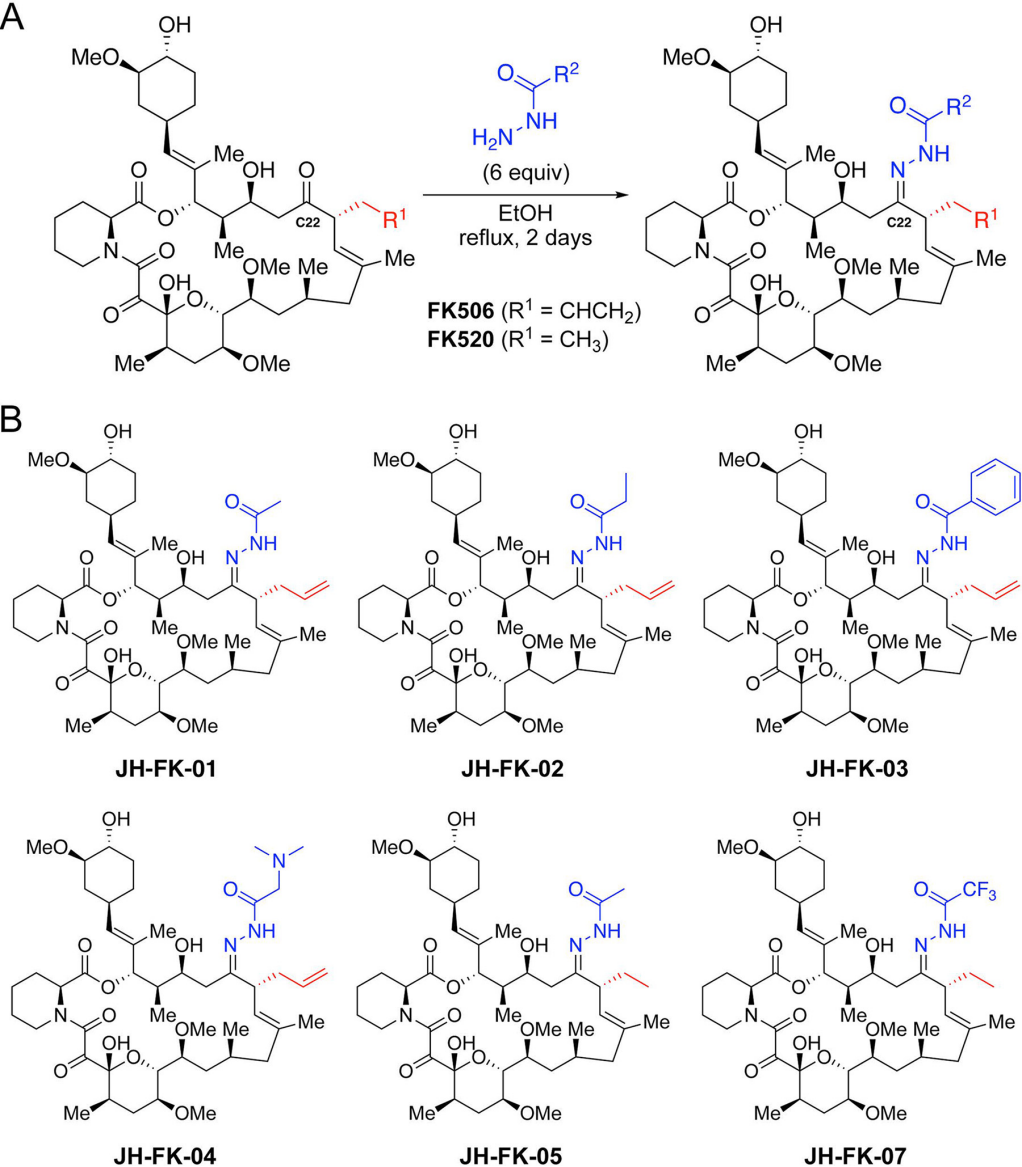
Chemical synthesis of novel FK506 and FK520 analogs. (A) One-step synthesis for C-22-modifed analogs from FK506 or FK520 as the starting material. (B) Panel of six FK506/FK520 analogs with acylhydrazone groups at C-22. Acylhydrazones differ in the R^2^ group. Compounds JH-FK-01, JH-FK-02, JH-FK-03, and JH-FK-04 are derivatives of FK506. Compounds JH-FK-05 and JH-FK-07 are derivatives of FK520.

Our previous study demonstrated that C-22 modifications of FK506 can introduce fungal selectivity (33). To leverage the protein crystal structures of FKBP12 bound to both FK506 and FK520, we developed a one-step synthetic protocol for C-22 modification compatible with either starting material (Fig. 2A). The C-22 position of FK506 and FK520 can be selectively targeted through an easily scalable and high-yield condensation reaction with an acylhydrazone under reflux conditions. Inspired by the success of the first-generation FK506 analog APX879, we resynthesized this compound (JH-FK-01) and generated five additional C-22-modified compounds (Fig. 2B). Of these five second-generation analogs, three were derived from FK506 (JH-FK-02, JH-FK-03, and JH-FK-04) and two were derived from FK520 (JH-FK-05 and JH-FK-07). Each analog was validated with high-resolution mass spectrometry (HRMS) and ^1^H nuclear magnetic resonance (NMR) (Table S2 and Fig. S4).

10.1128/mbio.01049-22.4 FIG S4 ^1^H nuclear magnetic resonance (NMR) spectra of FK506/FK520 analogs. All six synthesized FK506/FK520 analogs were subjected to ^1^H NMR analysis. All integrations are indicated. Chemical structure for each compound analyzed is presented at the left. Download FIG S4, PDF file, 0.9 MB. Copyright © 2022 Hoy et al. 2022 Hoy et al. https://creativecommons.org/licenses/by/4.0/ This content is distributed under the terms of the Creative Commons Attribution 4.0 International license.

10.1128/mbio.01049-22.6 TABLE S2 High-resolution mass spectrometry (HRMS) data for FK506/FK520 analogs. Download Table S2, DOCX file, 0.01 MB. Copyright © 2022 Hoy et al. 2022 Hoy et al. https://creativecommons.org/licenses/by/4.0/ This content is distributed under the terms of the Creative Commons Attribution 4.0 International license.

### Second-generation FK506 and FK520 analogs display broad-spectrum antifungal activity.

MICs/minimum effective concentrations (MECs) were determined for each of the novel analogs against the three major human fungal pathogens, A. fumigatus, C. neoformans, and C. albicans. Although all six analogs showed reduced antifungal activity compared to that of FK506, they were each effective at inhibiting growth in broth microdilution assays (Table 1). Along with their robust immunosuppressive activity, FK506 and FK520 exhibited remarkably potent antifungal activity in the range of 0.016 to 0.05 μg/mL, which is far below the single-digit micrograms per milliliter MIC threshold typically necessary to translate to *in vivo* antifungal efficacy. Thus, analogs of FK506 and FK520 can tolerate up to 100-fold reduction in antifungal activity and still be considered for *in vivo* testing. For C. neoformans, MICs were the most uniform and ranged from 0.2 to 2 μg/mL, which is between 4- and 40-fold reduced for antifungal activity. C. albicans MICs ranged from 0.2 to 6.25 μg/mL, which is between 10- and 312.5-fold reduced for activity. Despite being one of the most difficult to treat fungal infections, all six analogs were active against A. fumigatus with MECs ranging from 1 to 2 μg/mL or a 62.5- to 125-fold reduction in activity.

**TABLE 1:** MIC/MECs for FK506, FK520, and their analogs

Drug candidate	MIC (μg/mL) against:		MEC (μg/mL) against:
	Cryptococcus neoformans (H99)^*a*^	Candida albicans (SC5314)^*b*^	Aspergillus fumigatus (Af293)
FK506	0.05	0.02	0.016
FK520	0.05	0.02	—^*c*^
JH-FK-01	1	0.4	1
JH-FK-02	2	1.6	2
JH-FK-03	1	0.8	1
JH-FK-04	2	6.25	2
JH-FK-05	1	0.8	2
JH-FK-07	0.2	0.2	1.25

a C. neoformans MICs determined at 37°C.

b C. albicans MICs determined in the presence of 1 μg/mL fluconazole.

c —, Not tested.

Two FK520 analogs, JH-FK-05 and JH-FK-07, maintained high levels of broad-spectrum antifungal activity across the three species, and JH-FK-07 had the lowest MICs for both C. neoformans and C. albicans. To assess the antifungal activity against other human fungal pathogens, we selected JH-FK-05 to test against a panel of pathogenic molds and drug-resistant A. fumigatus strains (Table S3). JH-FK-05 registered MECs as low as 1 μg/mL for some of these highly recalcitrant species, such as Mucor circinelloides, Rhizopus oryzae, and pan azole-resistant A. fumigatus (strain F14946).

10.1128/mbio.01049-22.7 TABLE S3 Antifungal activity of JH-FK-05 against molds. Download Table S3, DOCX file, 0.01 MB. Copyright © 2022 Hoy et al. 2022 Hoy et al. https://creativecommons.org/licenses/by/4.0/ This content is distributed under the terms of the Creative Commons Attribution 4.0 International license.

To confirm that JH-FK-05 was fungal FKBP12-dependent, we utilized a humanized A. fumigatus strain (the *akuB^Ku80^ h*FKBP12 strain) expressing a codon-optimized human *FKBP12* gene from the *Affkbp12* native locus. We have previously shown that this *h*FKBP12-expressing strain is completely resistant to FK506 and that the activity of FK506 in Aspergillus is dependent on the presence of an FKBP12 that can form the FKBP12-inhibitor-calcineurin complex (33). Similar to FK506, JH-FK-05 has no detectable antifungal activity against the *h*FKBP12-expressing A. fumigatus strain, suggesting that JH-FK-05 works via the same mechanism of action (Table S3).

### FK506 and FK520 analogs bind FKBP12 and form a ternary inhibitory complex with calcineurin.

To better understand the relationship between structure and activity of these analogs, we tested one FK506 analog, JH-FK-02, and one FK520 analog, JH-FK-05, for their binding dynamics to CnA and CnB by biolayer interferometry (BLI). Purified and biotinylated (Avi-tagged) FKBP12 from A. fumigatus and C. albicans were loaded onto streptavidin sensors in the presence of an inhibitor, followed by titration of the respective CnA-CnB-calmodulin complex, also in the presence of an inhibitor. Data were double referenced using parallel reference sensors and sample blanks. Steady-state and kinetic analyses confirm that the target complexes form in the presence of the analogs with affinities similar to those of the parent compounds, suggesting that the *in vivo* observations are due to inhibitory effects of the compound-mediated calcineurin-FKBP12 complexes (Fig. S2).

10.1128/mbio.01049-22.2 FIG S2 BLI sensorgrams for compound-mediated binding interactions between A. fumigatus and Candida albicans CnA-CnB-calmodulin ternary complex and biotinylated (Avi-tagged)-FKBP12. Experiments were performed using a constant compound concentration of 10 μM and various concentrations of analyte protein (CnA-CnB-calmodulin, 2,500 to 39.0625 nM by serial 1:1 dilution, plus a 0 nM sample blank). All data are double referenced using sample blanks and parallel reference sensors. Three sample replicates were acquired in each experiment (light gray). Analysis was performed using 1:1 models and global analysis (calculated model traces in red). Biolayer inferometry (BLI) kinetics and steady-state analysis results are summarized in the table (bottom). Kinetics data were analyzed using 1:1 models and global analysis. Steady-state data were analyzed using binding responses in the 290 to 295 s range of sample association. Download FIG S2, PDF file, 0.7 MB. Copyright © 2022 Hoy et al. 2022 Hoy et al. https://creativecommons.org/licenses/by/4.0/ This content is distributed under the terms of the Creative Commons Attribution 4.0 International license.

### Some second-generation FK506 and FK520 analogs show increased fungal specificity.

**FIG 3:**
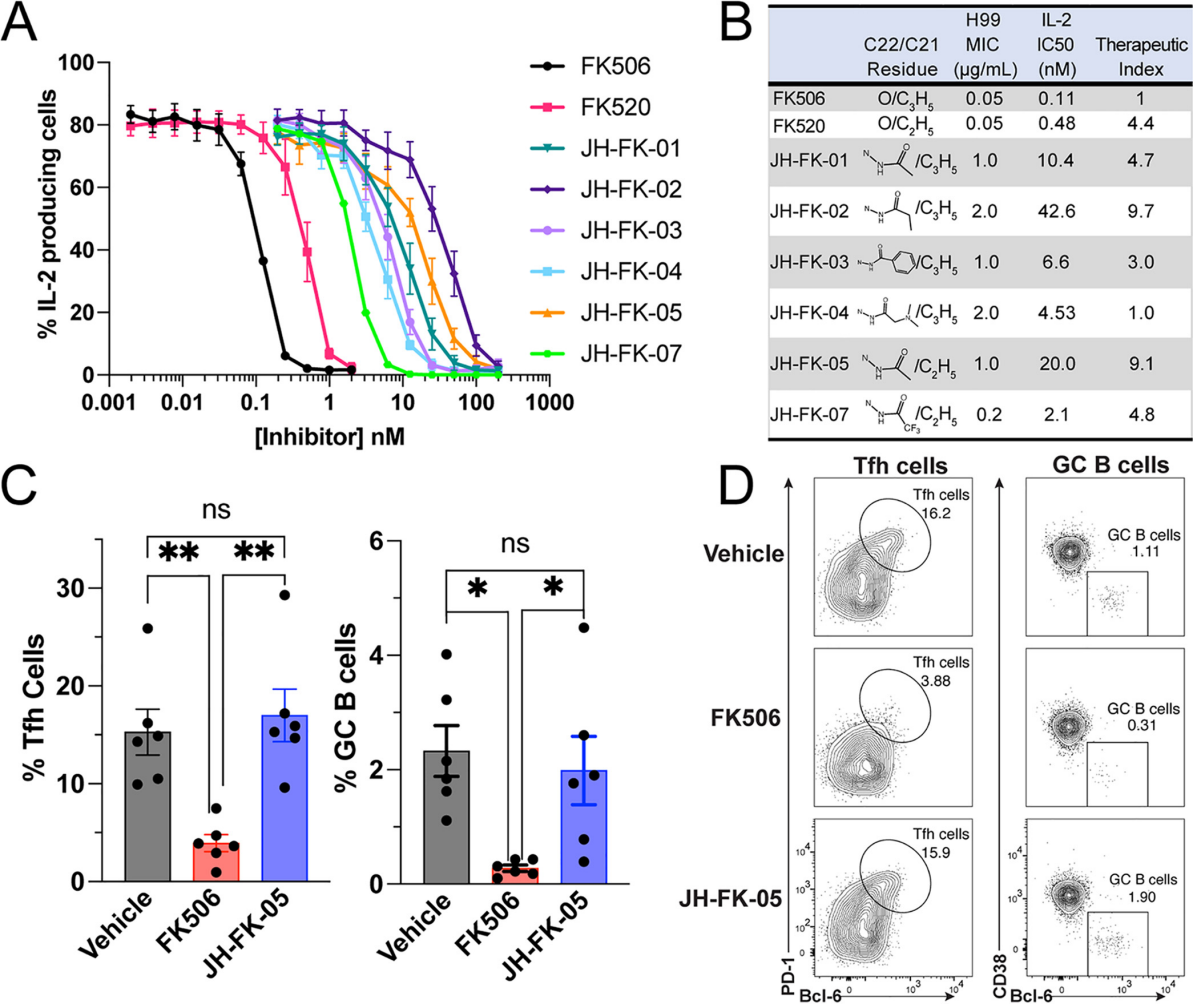
JH-FK-05 shows reduced immunosuppressive activity *in vitro* and *in vivo* and demonstrates fungal specificity. (A) *In vitro* immunosuppressive activity of calcineurin inhibitors was measured in a primary murine T-cell model. Dose response curves were generated from IL-2 expression in cells exposed to increasing concentrations of inhibitors. All analogs were reduced for immunosuppression compared to controls FK506 (black) and FK520 (pink). Error bars indicate standard error of the mean (SEM). (B) Therapeutic index scores for FK506/FK520 analogs were calculated by comparing the ratio of antifungal (MIC for C. neoformans) to immunosuppressive activity (IL-2 IC_50_) to the same ratio for FK506. FK506 therapeutic score was set to 1.0, and increasing scores indicate increasing fungal specificity. (C) *In vivo* immunosuppression of FK506 and JH-FK-05 was measured in a murine model of a T cell-dependent response. Animals were treated daily via i.p. injection with vehicle, FK506 (5 mg/kg), or JH-FK-05 (60 mg/kg) beginning on day −1. NP-OVA was administered subcutaneously on day 0 to promote T cell-dependent proliferation of T follicular helper cells (Tfh) and germinal center B (GC B) cells. The proportions of proliferated Tfh cells and GC B cells were measured in animal lymph nodes on day 7. Tfh and GC B cells were measured from TCRβ^+^ CD4^+^ CD44^hi^ cells and TCRβ^−^ CD19^+^ cells, respectively. (D) Representative flow cytometry plots are presented from individual animals in each treatment group. Gating indicates a region containing Tfh cells (left, oval) or GC B cells (right, square). Error bars indicate standard deviation. For all plots, **, *P* < 0.01; *, *P* < 0.05.

The potent immunosuppressive activity of FK506 and FK520 is what precludes these highly antifungal compounds from being utilized clinically to treat invasive fungal infections. Therefore, it is essential to understand the level of immunosuppressive activity for each of these analogs to determine if these C-22 modifications improve the fungal selectivity. Utilizing a primary murine T-cell model, we assessed IL-2 expression following growth in the presence of calcineurin inhibitors (Fig. 3A). Naive, primary CD4^+^ T cells were collected from mice and grown for 72 h in the presence of FK506, FK520, or an analog. Following stimulation with phorbol 12-myristate 13-acetate (PMA) and ionomycin, the proportion of cells producing IL-2, a key calcineurin-dependent cytokine, was measured with flow cytometry. All compounds generated a dose-dependent reduction in IL-2 production. FK506 and FK520 demonstrated the highest level of IL-2 inhibition, and the effect of FK520 on IL-2 production was slightly reduced compared to that of FK506. JH-FK-02 and JH-FK-05 were the least immunosuppressive second-generation analogs, with 50% inhibitory concentration (IC_50_) values of 42.6 nM (>380-fold increase compared to FK506) and 20.0 nM (>180-fold increase compared to FK506), respectively (Fig. 3A).

To assess the therapeutic potential for these compounds, it was important to develop an approach to score and rank them based on antifungal and immunosuppressive activities. The therapeutic index score (TI_score_) is a ratio of the immunosuppressive fold change to the antifungal fold change, with all values relative to the baseline FK506 activity (Fig. 3B). A TI_score_ of >1.0 indicates an increase in fungal specificity because the fold change of the immunosuppressive reduction is greater than the antifungal activity reduction. As an example, JH-FK-05 is 20-fold reduced for antifungal activity but about 180-fold reduced for immunosuppression. Therefore, the TI_score_ of JH-FK-05 is 9.1 (TI_score_ = 182/20). The top two analogs were JH-FK-02 (TI_score_ = 9.7) and JH-FK-05 (TI_score_ = 9.1). Due to the similar TI_score_, JH-FK-05 was prioritized to advance to animal trials on the merit of more pronounced antifungal activity compared to JH-FK-02.

### JH-FK-05 is nonimmunosuppressive in a murine model of T cell-dependent response.

Based on the *in vitro* evidence of reduced immunosuppressive activity of JH-FK-05, we hypothesized that this reduction would translate into reduced immunosuppression in an *in vivo* model. Our murine model for immunosuppression measures the T cell-dependent proliferation of two immune cell subtypes in response to immunization with an antigen. Animals were treated daily via intraperitoneal (i.p.) injection with vehicle, 5 mg/kg FK506, or 60 mg/kg JH-FK-05, beginning on day −1 and ending on day 7. JH-FK-05 treatment was tolerated at a dose as high as 60 mg/kg, and the final dose of JH-FK-05 was selected based on the maximum compound solubility (data not shown). On day 0, animals were immunized with NP-OVA to stimulate T cell-dependent signaling that would result in proliferation of T follicular helper (Tfh) cells and germinal center (GC) B cells. Animals were sacrificed on day 7, and the proportions of Tfh and GC B cells in the lymph nodes were measured. Treatment with 5 mg/kg of FK506 significantly reduced the proliferation of both Tfh and GC B cells (Fig. 3B). However, despite the much larger daily dose of 60 mg/kg, JH-FK-05 treatment did not reduce the presence of either cell type and was statistically indistinguishable from the vehicle treatment. Representative flow cytometry plots capture the lack of both immune cell subtypes in the FK506-treated animals only (Fig. 3D). In addition to its lack of immunosuppressive activity *in vivo*, JH-FK-05 was well tolerated by animals throughout the course of treatment.

### JH-FK-05 reduces pulmonary and brain fungal burden in models of cryptococcosis.

**FIG 4:**
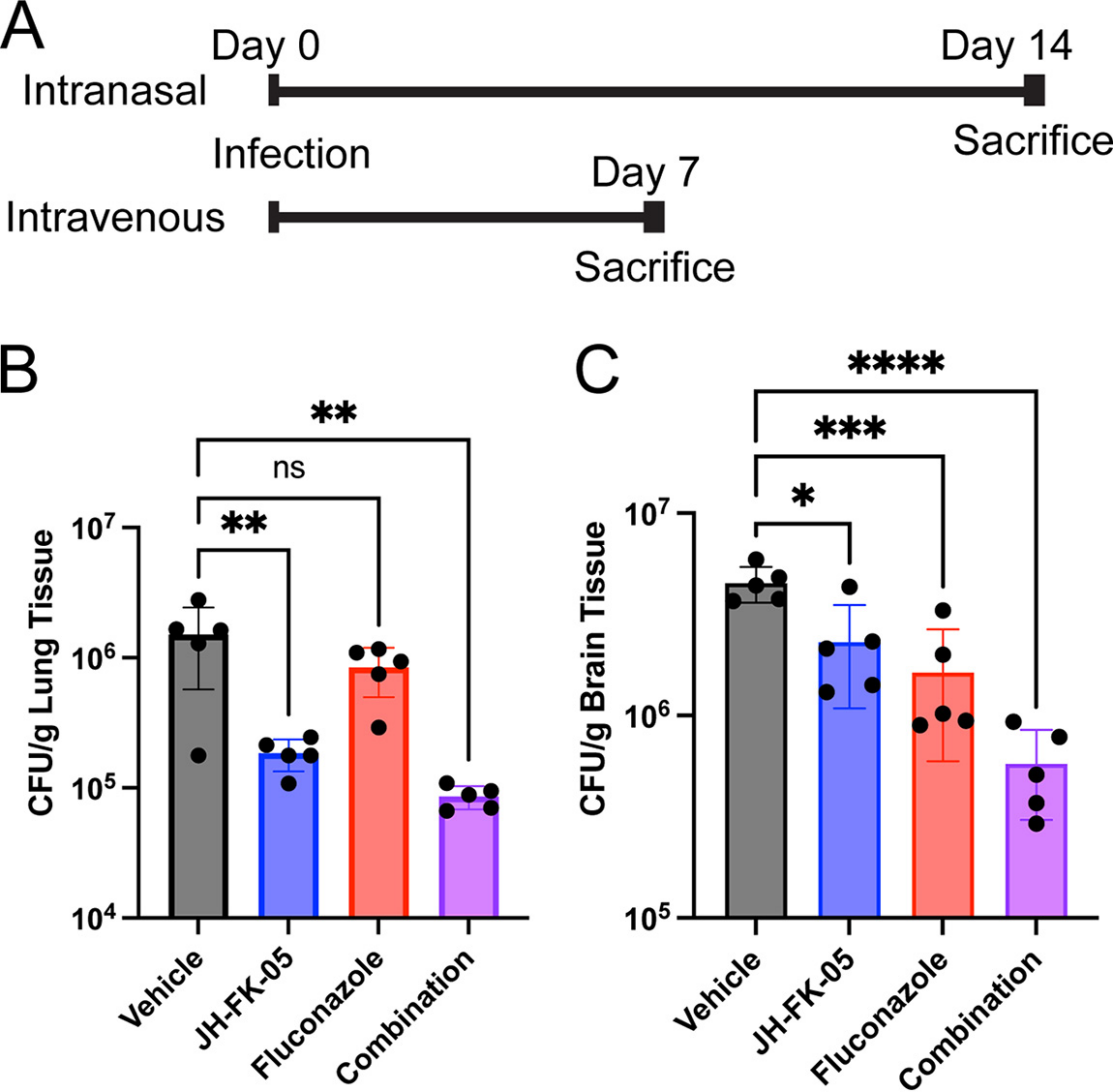
JH-FK-05 treatment reduces organ fungal burden in multiple models of C. neoformans infection. (A) Experimental timelines for intranasal and intravenous models of C. neoformans infection. For both models, treatment groups included vehicle (gray), JH-FK-05 60 mg/kg (blue), fluconazole 12 mg/kg (red), and combination (purple). Treatment began on day 0 via i.p. injection and continued daily until the indicated endpoint. Each treatment group contained 5 female A/J mice. (B) Lung tissue fungal burdens from animals in the intranasal model. (C) Brain tissue fungal burdens from animals in the intravenous model. For all plots, ****, *P* < 0.0001; ***, *P* < 0.001; **, *P* < 0.01; *, *P* < 0.05. Error bars indicate standard deviation.

The high *in vitro* antifungal activity of JH-FK-05 presented promising evidence that it might effectively reduce fungal burden in the context of murine infection. We employed two models of C. neoformans infection to test this hypothesis (Fig. 4A). First, in the intranasal model, animals were anesthetized and infected with 10^5^ cells of wild-type H99 C. neoformans via instillation in the animal’s nares. These animals were monitored for 14 days and then sacrificed. This model tests the efficacy of JH-FK-05 to treat a pulmonary infection, which is the most common manifestation of early-stage cryptococcosis in patients. Second, the intravenous (i.v.) model approximates the disseminated stage of disease. Animals were infected with 10^4^ cells via injection into the lateral tail vein. Due to the advanced nature of the model and the rapidly disseminated disease, animals were sacrificed at 7 days and found to have a substantial central nervous system (CNS) fungal burden. In both models, animals were treated once daily via i.p. injection beginning 3 h after infection with either vehicle, 60 mg/kg JH-FK-05, 12 mg/kg fluconazole, or a combination of the two monotherapies. Fluconazole is a widely used antifungal drug targeting ergosterol biosynthesis for cell membrane integrity. Previous work has shown that calcineurin inhibitors are synergistic with fluconazole (36, 37). Additionally, the first-generation FK506 analog, APX879, benefits from this synergistic interaction when analyzed *in vivo* in combination therapy with fluconazole in the pulmonary cryptococcosis model (33).

Like the immunosuppressive murine model, animals tolerated JH-FK-05 treatment for the full treatment period. Analysis of lung tissue collected from animals at day 14 showed that JH-FK-05 treatment alone reduced fungal burden by nearly 10-fold compared to that in vehicle-treated animals (Fig. 4B). The subtherapeutic dose of fluconazole alone did not reduce the fungal burden but combination therapy with JH-FK-05 significantly reduced the lung fungal burden by over 10-fold. To assess if JH-FK-05 treatment could display therapeutic levels of activity after the infection had fully disseminated, the intravenous model was employed. A modest but significant 2-fold reduction (*P* < 0.05) in brain fungal burden was detected in animals treated with JH-FK-05 alone (Fig. 4C). However, in combination, JH-FK-05 and fluconazole treatment resulted in a nearly 10-fold reduction in fungal burden after only 7 days of treatment. This demonstrates that not only can JH-FK-05 treat an aggressive pulmonary-stage infection but it can cross the blood-brain barrier to directly treat a disseminated CNS infection. This is in accord with previous studies and the known pharmacodynamic properties of FK506, which is known to cross the blood brain barrier (17). Together, these results demonstrate that JH-FK-05 is effective at reducing fungal burden in multiple stages of C. neoformans infection, both as a monotherapy and in combination with the FDA-approved antifungal drug fluconazole.

### Treatment with JH-FK-05 in murine C. neoformans infection extends survival.

**FIG 5:**
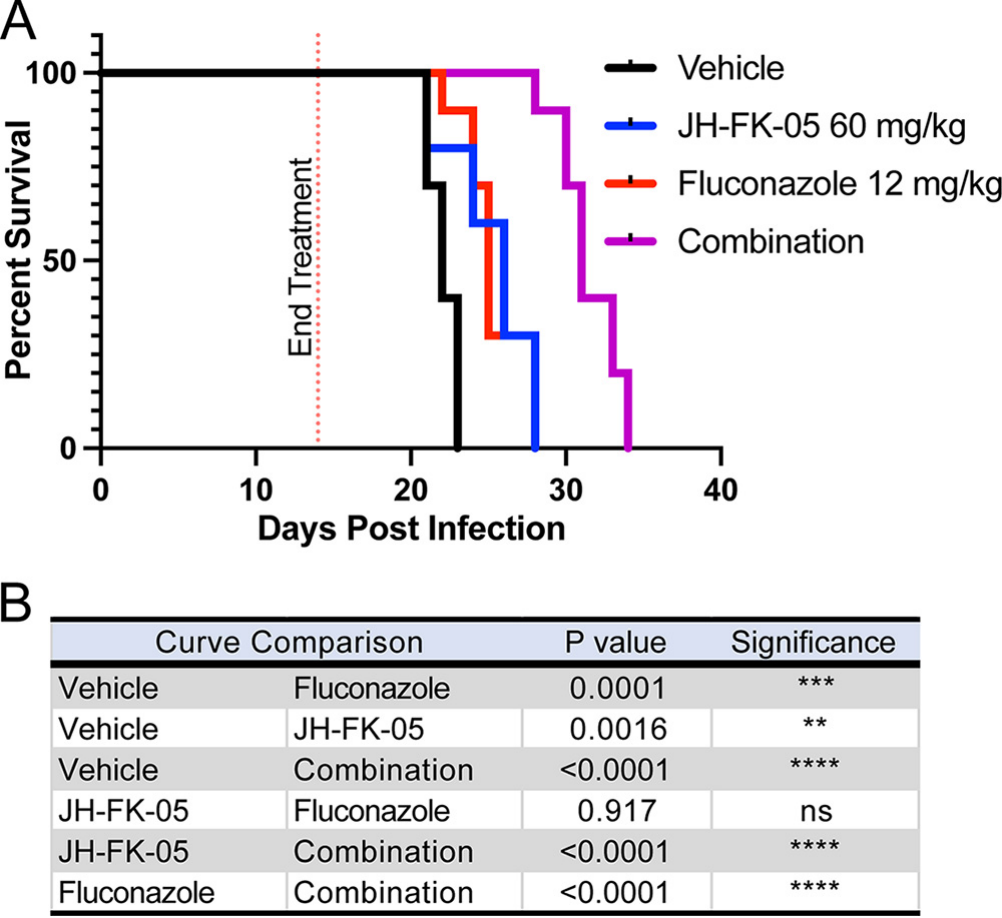
JH-FK-05 treatment extends animal survival in murine model of cryptococcosis. (A) Survival plot of animals infected with C. neoformans H99 through intranasal instillation. Treatment groups included vehicle (black), JH-FK-05 60 mg/kg (blue), fluconazole 12 mg/kg (red), and combination (purple). Animals received treatment via i.p. injection daily for 14 days, and animals were monitored for health following treatment termination. Each treatment group consisted of 10 A/J female mice. (B) Survival curve pairwise comparisons by log-rank (Mantel-Cox) test.

To verify if this reduction seen in the fungal burdens of JH-FK-05 and combination-treated animals could translate into a meaningful therapeutic effect, the survival of animals infected via the intranasal route was assessed (Fig. 5A). As in the fungal burden model, these animals received daily treatment for only 14 days. Following the termination of treatment, animals were monitored for signs of decline, lethargy, and ultimately, moribundity. Untreated animals in this model typically survive for about 3 weeks, and similar results were observed here (vehicle treatment median survival, 22 days). Corroborating the results of the fungal burden study, a significant extension of survival for both JH-FK-05 (median survival, 26 days) and fluconazole (median survival, 25 days) was observed. As with the fungal burden analysis, combination of JH-FK-05 and fluconazole had a potent therapeutic effect and extended survival of these animals to a maximum of 34 days (median survival, 31 days). Both monotherapy survival curves were significantly different from the combination therapy, suggesting an additive or synergistic effect *in vivo* (Fig. 5B).

### Molecular dynamic simulations identify novel JH-FK-05 residues for future medicinal chemistry.

**FIG 6:**
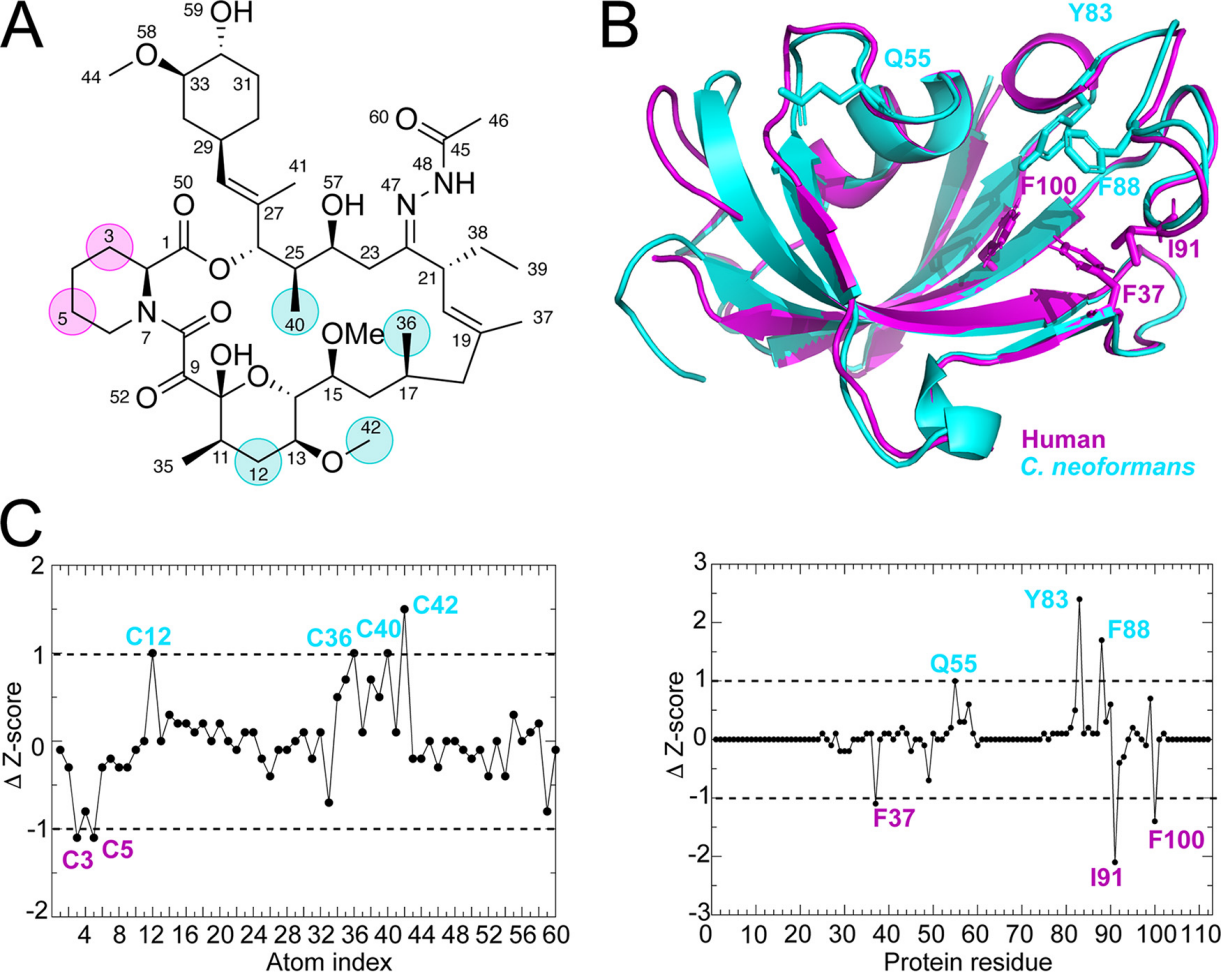
Ligand atoms and protein residue contacts observed in MD simulations. Analysis of the significance of the observed contacts of human FKBP12 (*h*FKBP12) and C. neoformans FKBP12 (*cn*FKBP12) with JH-FK-05. (A) Stick representation of JH-FK-05 labeled for atoms making more significant contacts to *h*FKBP12 (magenta circles) and C. neoformans (cyan circles). (B) Residues of *h*FKBP12 (magenta) and C. neoformans (cyan) making more significant interactions with the ligand are shown in stick format on the ribbon protein structures. (C) Z-score graphs. Black circles represent Z-scores of C. neoformans FKBP12 contacts minus *h*FKBP12 contacts. More significant contacts for C. neoformans (ΔZ-score > 1) are labeled in cyan, while contacts more significant for the human protein (ΔZ-score < 1) are labeled in magenta.

Although JH-FK-05 has demonstrated robust *in vitro* and *in vivo* antifungal activity, additional improvements can be made to elevate the TI_score_ of this compound. To address this, molecular dynamic (MD) simulations of JH-FK-05 physical interactions with human versus fungal FKBP12 were conducted to identify contact residues that are more important for either species (Fig. 6) (35). A series of 500-ns MD simulations between *h*FKBP12 and JH-FK-05 or C. neoformans FKBP12 and JH-FK-05 were simulated, and differential contacts between JH-FK-05 and the two FKBP12 proteins were observed. Simulation stability was monitored with alpha carbon (C-α) RMSD, radius of gyration, and center of mass distance (Fig. S3). Both the significant protein residues and the JH-FK-05 molecular residues that were identified are labeled on the respective structures (Fig. 6 and Table S4). There were 4 residues of JH-FK-05 that were more significant for C. neoformans contact (Fig. 6, cyan label). Interestingly, there were only 2 residues that were more significant for the JH-FK-05 and human FKBP12 (*h*FKBP12) interaction (Fig. 6, magenta labels). These residues are both in the C-2–N-7 ring of the molecule and represent potential future target areas for medicinal chemistry to introduce further fungal specificity to JH-FK-05 and design a new generation of analogs.

10.1128/mbio.01049-22.3 FIG S3 Analysis of 500-ns molecular dynamic (MD) simulation stability. MD simulations were run for JH-FK-05 binding either human FKBP12 (*h*FKBP12) or C. neoformans FKBP12. (Top) Figure plotting the alpha carbon (C-α) root mean square deviation (RMSD) for each MD simulation over the length of the simulation. (Middle) Figure plotting the radius of gyration (Rg) for each MD simulation over the length of the simulation. (Bottom) Figure plotting the center of mass (COM; the three-dimensional [3D] point of mass balance for each monomer) between the protein and ligand for each MD simulation over the length of the simulation. MD simulation 3 of *h*FKBP12-JH-FK-05 was removed from the analysis due to unstable COM values observed during the length of the simulation. Download FIG S3, PDF file, 0.3 MB. Copyright © 2022 Hoy et al. 2022 Hoy et al. https://creativecommons.org/licenses/by/4.0/ This content is distributed under the terms of the Creative Commons Attribution 4.0 International license.

10.1128/mbio.01049-22.8 TABLE S4 Atom index for JH-FK-05 molecular dynamic (MD) analysis. Download Table S4, DOCX file, 0.02 MB. Copyright © 2022 Hoy et al. 2022 Hoy et al. https://creativecommons.org/licenses/by/4.0/ This content is distributed under the terms of the Creative Commons Attribution 4.0 International license.

## DISCUSSION

In this study, a panel of second-generation FK506 and FK520 analogs were screened and JH-FK-05 was identified as a promising antifungal candidate that targets fungal calcineurin with a high degree of specificity. We also report the first fungal FKBP12-FK520 protein crystal structure at a high resolution of 1.7 Å. Much of this work was made possible by the structure-guided design and development of a first-generation FK506 analog, APX879 (33). Crystal structures of fungal FKBP12 proteins revealed a key conserved difference in the 80s loop that may promote fungal selectivity in molecules modified at the C-22 position of FK506. The A. fumigatus FKBP12-FK520 structure clearly shows that both the C-22 and C-21 residues of FK520 approach the Phe88 residue (Fig. 1C). This proximity and potential for a steric clash suggested that modifications at the C-22 position on both FK506 and FK520 could introduce a high degree of selectivity.

Establishing the therapeutic window for these calcineurin inhibitors was an essential component of developing small molecules that were fungus-specific. Because FK506 and FK520 already have exceedingly high antifungal activity in addition to their immunosuppressive activity, the goal was to reduce the immunosuppression while preserving antifungal activity to shift the balance in favor of this therapeutic window. Both inhibitors have stronger antifungal activity against C. neoformans than the front-line treatments for cryptococcosis, amphotericin B (MIC 0.125 μg/mL) and fluconazole (3 μg/mL) (Table 1) (38, 39). Due to the elevated starting point for both of these compounds, we were able to afford a significant reduction in the antifungal activity seen in each of our analogs. Two compounds were identified, JH-FK-02 and JH-FK-05, that demonstrate a nearly 10-fold increase in therapeutic index score overall and are only 40- and 20-fold reduced for antifungal activity, respectively (Fig. 3B). These compounds also helped establish a “threshold” for prioritizing future compounds for advancement into animal models. Based on the high antifungal activity of FK506/FK520 and the reported MIC/MECs for clinically utilized antifungal drugs, we estimate that our analogs can tolerate up to a 100-fold reduction in antifungal activity and still perform well in infection models (38, 39).

JH-FK-05 was selected for further *in vitro* mold testing and *in vivo* animal models due to its higher degree of antifungal activity compared to that of JH-FK-02 (Fig. 3B). Remarkably, despite a daily dose at 12 times that of FK506, there was no detectable change in the T cell-dependent response compared to that in vehicle-treated animals (Fig. 3C). This shift in the immunosuppressive activity facilitated the *in vivo* dosing of JH-FK-05 at a high level of 60 mg/kg and was limited primarily by solubility and not by tolerability. Comparatively, in the same murine infection models, we showed that a lower, 20 mg/kg dose of FK506 is lethal to animals (33). Although not compared directly in this study, JH-FK-05 represents a significant reduction in toxicity and improvement in efficacy to be within the therapeutic window. Pharmacokinetic investigation of JH-FK-05 treatment could lead to an optimization of the dosage frequency and total dose in the future. Additionally, histopathological analysis of infected tissue will determine the level of residual fungal growth and assess the recruitment of immune cells to the site of infection. Due to the high degree of selectivity observed with JH-FK-05, it is possible that treatment will not be limited by toxicity but instead be limited by the solubility and delivery route of the treatment. Fortunately, both FK506 and FK520 are orally active compounds in both humans and rodents (40). Additional investigation will determine if JH-FK-05 can be orally administered, but the pharmacokinetic properties of the parent molecule FK520 suggests that this is likely to be the case. The development of an orally active antifungal compound with broad-spectrum activity would be a major addition to the currently limited antifungal armamentarium (4).

We utilized two different models of Cryptococcus infection to address the key stages of infection experienced by patients. The intranasal infection model is similar to the progression of disease observed in most patients that begin with a pulmonary infection. The intravenous model allowed us to test if JH-FK-05 can treat an already disseminated infection and exert activity across the blood-brain barrier (BBB) (Fig. 4). FK506 is known to cross the BBB, so it was validating to observe that JH-FK-05 reduced fungal burden in the brain of intravenously (i.v.) infected mice (17). CNS engagement and antifungal activity across the BBB are critical factors in developing treatments with therapeutic potential for the treatment of cryptococcosis. There is an increasing need for treatments that can directly address the CNS stage of infection (4). Without early intervention, the majority of pulmonary C. neoformans infections will disseminate to become aggressive CNS infections. In these cases, high rates of mortality in all groups of patients diagnosed with cryptococcal meningitis is a direct result of treatments failing to act on the disseminated versus the localized pulmonary disease stage (41, 42). JH-FK-05 represents a promising opportunity to address both early- and late-stage C. neoformans infections.

In each of our infection models, we demonstrated that combination treatment with fluconazole increased the potency of JH-FK-05 (Fig. 4 and 5). In fact, the gold standard of treatment for C. neoformans infections is a combination therapy of amphotericin B and 5-fluorocytosine (43). Other antifungal combination therapies that have shown efficacy in the clinic include fluconazole-amphotericin B (candidemia) and caspofungin-amphotericin B (mucormycosis) (44). Due to rising rates of antifungal drug resistance, combination therapies that generate additive or synergistic interactions are essential in treatment plans. We suggest that the efficacy of JH-FK-05 as both a monotherapy and a combination therapy with a clinical antifungal drug is an exciting development and a promising indicator that this approach could lead to a treatment with substantial clinical impact.

Multiple groups have developed biosynthetic analogs of FK506 by deleting genes in the FK506 biosynthetic cluster of the *Streptomyces* organism (45). After the macrolide base of FK506 is produced by polyketide synthase (PKS) steps, several enzymes introduce post-PKS modifications to the macrolide ring. Deletion of genes encoding these enzymes (*fkbM*, *fkbD*) resulted in different FK506 analogs with various degrees of immunosuppressive and antifungal activity (37). The scalability of this approach is extremely promising. Although this strategy is ultimately limited in rational design, future combinatorial synthetic approaches can unlock the medicinal chemistry at sites across the FK506 molecule. Most biosynthetic analogs are compatible with our procedure to modify the C-22 position of FK506/FK520 and could be utilized as starting material to synthesize a new generation of analogs through a combinatorial biosynthetic-medicinal chemistry strategy.

Future second-generation FK520 analog development will also be informed by MD simulations that identify target residues on the molecule that are more important for fungal versus mammalian interaction. Data from MD simulations performed with JH-FK-05 binding human versus C. neoformans FKBP12 revealed multiple residues that increased fungal specificity (Fig. 6). This approach has successfully identified areas of the first-generation FK506 analog, APX879, that are enriched for contact with only the human FKBP12 (35). Modifications made to JH-FK-05 could further increase its TI_score_ and drive the therapeutic potential even further. This approach can be applied to any additional analogs that are developed in the future as potential starting material for combinatorial synthetic strategies at both C-22 and C-21 positions. Our preclinical success with JH-FK-05 indicates that iterative and structure-informed design of FK506 analogs is a promising strategy to develop fungus-specific calcineurin inhibitors as therapeutics.

## MATERIALS AND METHODS

### Protein production.

See Text S1 in the supplemental material for full methods for protein production and purification for X-ray crystallography. A. fumigatus FKBP12 P90G was expressed and purified as previously described (33).

10.1128/mbio.01049-22.9 TEXT S1 Methods of protein production. Download Text S1, DOCX file, 0.02 MB. Copyright © 2022 Hoy et al. 2022 Hoy et al. https://creativecommons.org/licenses/by/4.0/ This content is distributed under the terms of the Creative Commons Attribution 4.0 International license.

### Complexes of *A. fumigatus* CnA-CnB + *A. fumigatus* FKBP12-P90G with FK506 and *H. sapiens* CnA-CnB + Bio-*H. sapiens* FKBP12 with FK520.

Complexes were formed by first mixing FKBP12 with 1.5 × FK506/FK520 at 4°C for 30 min followed by addition of CnA-CnB. The combined sample was incubated for an additional 30 min at 4°C, then injected onto a Superdex 200 column (GE Healthcare) equilibrated in 25 mM Tris (pH 8.0), 200 mM NaCl, 1 mM Tris(2-carboxyethyl)phosphine hydrochloride (TCEP), and 5 mM CaCl_2_. Fractions containing the 1:1 complex were pooled and concentrated to 20 to 24 mg/mL.

### Structure determination by X-ray crystallography.

A. fumigatus FKBP12 was preincubated at room temperature for 30 min with 1.6 mM FK520 (100 mM dimethyl sulfoxide [DMSO] stock, compound to protein ratio of 1.26) prior to being placed in sparse matrix crystallization trials using sitting drop vapor diffusion at 18.2 mg/mL. Crystallization trays were stored at 14°C, and crystals grew over the course of 4 months. Crystals were grown in JCSG+ (Rigaku Reagents) condition A1 containing 0.2 M lithium sulfate (pH 4.5) and 50% vol/vol polyethylene glycol (PEG) 400, which also acted as a cryoprotectant during harvest. The data set was collected on 16 September 2021 at the synchrotron Advanced Photon Source (APS) beamline 21-ID-F on an MAR 300 charge-coupled device (CCD) X-ray detector. Two copies of the compound-bound FKBP12 were placed per asymmetric unit (PDB ID 7U0S). The structure was solved by molecular replacement using a previously solved structure of A. fumigatus FKBP12 bound to FK506 (PDB ID 5HWC). Molecular graphics and analyses performed with UCSF Chimera, developed by the Resource for Biocomputing, Visualization, and Informatics at the University of California, San Francisco, with support from NIH P41-GM103311 (46).

### Crystallization conditions of *A. fumigatus* CnA-CnB + *A. fumigatus* FKBP12-P90G with FK506 and *H. sapiens* CnA-CnB + Bio-*H. sapiens* FKBP12 with FK520.

Proteins were placed in sparse matrix crystallization trials using sitting drop vapor diffusion. Crystallization trays were stored at 14°C. Crystals of *A. fumigatus* CnA-CnB (*Af*CnA-CnB) + *Af*FKBP12-P90G with FK506 grew after 1 month in JCSG+ condition C6 (Rigaku Reagents) containing 0.1 M sodium phosphate dibasic/citric acid) (pH 4.2) and 40% (vol/vol) PEG 300 (also cryoprotectant). Crystals of *H. sapiens* CnA-CnB (*Hs*CnA-CnB) + Bio-*Hs*FKBP12 with FK520 grew after 1 day in MCSG-1 condition C5 (Microlytics) containing 0.2 M magnesium acetate and 20% (wt/vol) PEG 3350 (cryopreserved in 20% ethylene glycol). Data sets were collected on 10 December 2020 at the synchrotron APS beamline 21-ID-F on an MAR 300 CCD X-ray detector. A single copy of each complex was placed per asymmetric unit (*Hs*CnA-CnB + Bio-*Hs*FKBP12 with FK520 [PDB ID 7U0T]; *Af*CnA-CnB + *Af*FKBP12-P90G with FK506 [PDB ID 7U0U]). The structure of *Hs*CnA-CnB + Bio-*Hs*FKBP12 with FK520 was solved by molecular replacement using a previously solved structure of Bos taurus CnA, CnB, and FKBP12 bound to FK506 (PDB ID 1TCO), and the structure of *Af*CnA-CnB + *Af*FKBP12-P90G with FK506 was solved by molecular replacement using a previously solved structure of A. fumigatus CnA, CnB, and FKBP12 bound to FK506 (PDB ID 6TZ7).

Diffraction data were reduced and scaled with XDS/XSCALE (47). Structures were solved by molecular replacement using the program Phaser in the Phenix program suite (48). Structures were refined using iterative cycles of Translation/Libration/Screw (TLS) and restrained refinement with Phenix Refine and model building using COOT (49). The final structures were validated using MolProbity and deposited in the Protein Data Bank (50–52). Diffraction data and refinement statistics are listed in Table S1. Diffraction images are available at the Integrated Resource for Reproducibility in Macromolecular Crystallography (http://proteindiffraction.org/) (53, 54).

### Synthesis of FK506 and FK520 analogs.

FK506 and FK520 monohydrate starting materials were purchased from APIChem Technology (catalog no. AC-32477 and AC-5267). FK506 and FK520 analogs were prepared as follows. To a solution of FK506 or FK520 in anhydrous ethanol (EtOH; 0.1 M) was added acylhydrazine (6.0 equivalent) at 25°C. The resulting mixture was refluxed under N_2_ atmosphere. After stirring for 36 to 48 h, the reaction mixture was cooled to 25°C and concentrated *in vacuo*. The resulting mixture was diluted with ethyl acetate (EtOAc) and H_2_O. The layers were separated, and the aqueous layer was extracted with EtOAc. The combined organic layers were washed with brine, dried over anhydrous Na_2_SO_4_, and concentrated *in vacuo*. The residue was purified by silica gel column chromatography. All compounds were confirmed by HRMS and ^1^H NMR (Table S2 and Fig. S4).

### Antifungal susceptibility testing.

*In vitro* antifungal activities of FK506, FK520, and analogs were assessed in RPMI 1640 (Sigma-Aldrich) for all strains tested. MICs/minimum effective concentrations (MECs) for each drug were measured using modified Clinical and Laboratory Standards Institute (CLSI) M38-A2 and M27-A3 standard *in vitro* antifungal susceptibility protocols. Broth microdilution assays for MIC/MEC assessment were performed as described previously (33). All antifungal assays with C. neoformans were performed at 37°C. Antifungal susceptibility testing for C. albicans was performed in the presence of a uniform 1 μg/mL concentration of fluconazole to sensitize the organism to calcineurin inhibition.

### Biolayer interferometry binding experiments.

Biolayer interferometry (BLI) experiments were completed using either A. fumigatus or C. albicans FKBP12 and CnA/CnB/calmodulin complexes, along with compounds FK506, APX879, FK520, JH-FK-02, and JH-FK-05. Initial buffer (25 mM Tris, 200 mM NaCl, and 1 mM TCEP [pH 8.0]) was prepared using 1 M Tris-HCl (pH 8.0, catalog no. T1080; Teknova), 5 M sodium chloride (catalog no. S0251; Teknova), TCEP (catalog no. M115; Soltec Ventures, Inc.), and was readjusted to pH 8 before sterile filtering. Bovine serum albumin (BSA)-containing buffer was prepared by addition of BSA stock (30% in Dulbecco’s phosphate-buffered saline [DPBS], catalog no. A9576; Millipore Sigma) into the initial buffer to a final concentration of 1%. Compound-containing assay buffers were prepared by addition of compound stock (100 mM in DMSO-*d*_6_) into BSA-containing buffer to a final concentration of 10 μM. FKBP12 solutions were prepared using a two-step process in which FKBP12 was first diluted to 100 μg/mL with BSA-containing buffer, followed by dilution to 10 μg/mL with compound-containing assay buffer. Initial 2,500 nM CnA/CnB/calmodulin complexes were prepared by direct dilution of protein stock with compound-containing assay buffer. Serial 1:1 dilutions were then performed with compound-containing assay buffer to prepare the remaining samples in the series (full series: 2,500 nM, 1,250 nM, 625 nM, 312.5 nM, 156.25 nM, 78.125 nM, 39.0625 nM, and 0 nM [sample blank]). FKBP12 and CnA/CnB/calmodulin complexes were incubated overnight prior to experiments.

Experiments were performed on an Octet Red96e instrument (Sartorius) utilizing Octet Data Acquisition software version 11.1.1.19, equilibrated to 25°C. Streptavidin biosensors (SA, catalog no. 18-5019; Sartorius) were hydrated for a minimum of 30 min in compound-containing assay buffer prior to the start of each experiment. Each experiment consisted of two identical assays, each performed using separate biosensors—one using sensors loaded with FKBP12 (sample sensors), and the other using unloaded sensors (parallel reference sensors). Each assay consisted of the following steps: initial baseline (120 s, compound-containing assay buffer); FKBP12 loading (170 s for Aspergillus, 180 s for *Candida*); sample baseline (120 s, compound-containing assay buffer); CnA/CnB/calmodulin complex association (300 s); dissociation (900 s, same compound-containing assay buffer wells as in the sample baseline step); sample baseline/association/dissociation cycle 2 (same times and wells as in previous steps); sample baseline/association/dissociation cycle 3 (same times and wells as in previous steps). Data were acquired for FKBP12-loaded sensors first and parallel reference sensors second.

Data were processed and analyzed using ForteBio Data Analysis software version 11.1.3.10. Data were processed using the “double reference subtraction” option, with the *y* axis aligned to baseline (from 0.1 to 119.8 s), interstep correction aligned to dissociation, and Savitzky-Golay filtering active. Kinetics data were analyzed using the “association and dissociation curve fitting” option utilizing a 1:1 model. Fitting was performed using the “global (full)” option with “*R*_max_ unlinked by sensor” (the one exception being Aspergillus sample JH-FK-05, which would not process cleanly unless the “*R*_max_ linked” option was selected instead). The window of interest spanned the full association and dissociation steps of each experiment. Steady-state analysis was completed using the response average from 290.0 to 295.0 s of the association step (because some binding responses did not reach equilibrium).

### *In vitro* immunosuppressive activity testing.

Spleen and lymph nodes were collected from C57BL/6 mice and homogenized through a 40-μm filter. Pan-CD4^+^ T cells were enriched using a MagniSort mouse CD4 T-cell kit (eBioscience) according to the manufacturer’s protocol. Thereafter, CD4^+^ CD25^−^ CD44^lo^ CD62L^hi^ naive T cells were sorted by fluorescence-activated cell sorting (FACS). The naive CD4^+^ T cells were grown in Iscove’s modified Dulbecco’s medium (IMDM) supplemented with glutamine, penicillin, streptomycin, gentamicin, 2-mercaptoethanol, and 10% fetal bovine serum (FBS). Cells were then cultured on anti-hamster IgG-coated plates in the presence of hamster anti-CD3 epsilon and anti-CD28 antibodies (BD Biosciences), neutralizing anti-IL-4 antibody (eBioscience), recombinant IL-12 (10 ng/mL), and recombinant IL-2 (50 U/mL) for 72 h. During this period, cells were grown in the presence of 2× serially diluted FK506, FK520, or analog suspended in DMSO for 72 h. During the last 4 h of culture, phorbol 12-myristate 13-acetate (PMA; Sigma), ionomycin (Sigma), and GolgiStop (BD Biosciences) were added to the culture to facilitate detection of intracellular cytokines via flow cytometry.

Live cells were stained with fluorescein isothiocyanate (FITC)-conjugated anti-CD4 antibody (eBioscience) and fixable viability dye eFluor 506 (eBioscience) on ice, followed by fixation and permeabilization using the Foxp3 transcription factor staining kit (eBioscience). Intracellular staining was performed at room temperature using phycoerythrin (PE)-conjugated anti-IL-2 antibody (eBioscience), followed by analysis with a BD LSRFortessa X-20 flow cytometer.

### Preparation of compounds for *in vivo* studies.

For *in vivo* studies on immunosuppressive activity, FK506 was purchased from Duke Pharmacy as Prograf (tacrolimus; Astellas) and was used as a solution of 1 mg/mL. Vehicle was prepared as a sterile solution of 90% phosphate-buffered saline (PBS), 5% Kolliphor EL (catalog no. C5135; Sigma), and 5% ethanol. JH-FK-05 stock solution of 120 mg/mL was prepared as a powder dissolved in sterile 50% Kolliphor EL and 50% ethanol. For administration to animals, JH-FK-05 stock was diluted to 12 mg/mL and 10% Kolliphor-ethanol in sterile PBS. Fluconazole was purchased from Duke University Hospital Pharmacy as a sterile solution of fluconazole 2 mg/mL in PBS.

### *In vivo* immunosuppressive activity assessment of JH-FK-05.

Groups of 6 female C57BL/6 mice (Jackson Laboratory) were treated daily by intraperitoneal (i.p.) injection with about 100 μL vehicle, 5 mg/kg FK506, or 60 mg/kg JH-FK-05 beginning on day −1. On day 0 mice were immunized with NP-OVA in alum via subcutaneous injection. Mice were treated and monitored daily until they were sacrificed on day 7. Draining lymph nodes were harvested on day 7, and the populations of T follicular helper cells and GC B cells were analyzed by flow cytometry. Tfh and GC B cells were measured from T Cell Receptor β (TCRβ^+^) CD4^+^ CD44^hi^ cells and TCRβ^−^ CD19^+^ cells, respectively.

### C. neoformans cell preparation for murine infection.

Wild-type H99 C. neoformans cells were grown overnight at 30°C in a roller drum in yeast extract-peptone-dextrose (YPD) medium. Cells were pelleted at 3,000 rpm and washed 3 times with sterile PBS. H99 cells were counted with a hemacytometer and diluted to 2 × 10^6^ cells/mL in PBS for the pulmonary model and 2 × 10^5^ cells/mL in PBS for the disseminated model.

### Intranasal model of pulmonary cryptococcosis.

Female A/J mice (3 to 4 weeks old; Jackson Laboratory) were anesthetized utilizing an isoflurane chamber. While anesthetized, 50 μL of 2 × 10^6^ cells/mL suspension was dripped onto the nares of mice, allowing them to inhale the full inoculum. Treatment began 3 h following infection with i.p. dosing of vehicle, 60 mg/kg JH-FK-05, 12 mg/kg fluconazole, or combination. Animals were treated daily for 14 days. After 14 days, animals were either sacrificed for fungal burden analysis or monitored for health and survival until reaching a humane endpoint. Survival analysis was plotted and performed in GraphPad Prism 9. Statistical significance was determined using log-rank (Mantel-Cox) tests.

### Intravenous model of disseminated cryptococcosis.

Male CD1 mice (3 to 4 weeks old; Charles River) were restrained in a 50-mL conical tube with holes at both ends for ventilation. Animal tails were warmed under a heat lamp to enhance vasodilation and sterilized with alcohol wipes. A 50-μL suspension of 2 × 10^5^ cells/mL was injected into the lateral tail vein with an insulin syringe. Treatment proceeded as described in the intranasal infection model until day 7, when animals were sacrificed for fungal burden analysis.

### Cryptococcus fungal burden analysis.

Mice were euthanized by CO_2_ inhalation at the predetermined experimental endpoint. Organs from each animal were harvested and homogenized in sterile PBS using steel beads (Qiagen) and a bead beater. Organ homogenate was serially diluted in PBS. A 100-μL aliquot of each dilution was plated on antibiotic plates (YPD, 50 μg/mL ampicillin, and 30 μg/mL chloramphenicol), incubated for 48 to 72 h at 30°C, and assessed for CFU. CFU per gram of organ was plotted using GraphPad Prism 9. Statistical significance was calculated using an ordinary one-way analysis of variance (ANOVA) with Tukey’s multiple-comparison test.

### Molecular dynamic simulations.

MD simulations were performed to provide a better representation of the protein’s conformational flexibility and to more accurately characterize the protein’s solution structure bound to JH-FK-05. The following crystal structures were used as the starting conformations: *h*FKBP12 bound to FK506 (PDB ID 1FKF) and *C. neoformans* FKBP12 (*cn*FKBP12) (PDB ID 6TZ8, with calcineurin A and B removed) (33, 55). JH-FK-05 small-molecule parameter and topology files were downloaded and created utilizing the Automated Topology Builder (ATB) and repository (56, 57). All molecular dynamic (MD) simulations were performed with the GROMACS 5.0.1 software package utilizing 6 CPU cores and one Nvidia Tesla K80 GPU (58). The single starting conformations used for all of the MD simulations were the X-ray-characterized crystal structures noted above. MD simulations were performed with the GROMOS54a7 force field and the flexible simple point-charge water model. The initial structures were immersed in a periodic water box with a dodecahedron shape that extended 1 nm beyond the protein in any dimension and were neutralized with counter ions. Energy minimization was accomplished through use of the steepest descent algorithm with a final maximum force below 100 kJ/mol/min (0.01-nm step size and cutoff of 1.2 nm for neighbor list, Coulomb interactions, and Van der Waals interactions). After energy minimization, the system was subjected to equilibration at 300 K and normal pressure for 1 ns. All bonds were constrained with the LINCS algorithm (cutoff of 1.2 nm for neighbor list, Coulomb interactions, and Van der Waals interactions). After temperature stabilization, pressure stabilization was obtained by utilizing a V-rescale thermostat to hold the temperature at 300 K, and a Berendsen barostat was used to bring the system to a pressure of 10^5^ Pa. Production MD calculations (500 ns) were performed under the same conditions, except that the position restraints were removed, and the simulation was run for 500 ns (cutoff of 1.1, 0.9, and 0.9 nm for neighbor list, Coulomb interactions, and Van der Waals interactions). These MD simulations were repeated 6 times. Alpha carbon (C-α) root mean square deviation (RMSD), radius of gyration (Rg), and center of mass (COM) all confirmed the stability and accuracy of the MD simulations by stabilizing after a 100-ns equilibration period (in most cases), allowing for analysis of the last 400 ns of the simulations. Only one of the 12 calculations (*h*FKBP12:JHFK05 complex MD sim no. 3) was rejected from the analysis due to unstable C-α RMSD, Rg, and/or COM (Fig. S3). GROMACS built-in and homemade scripts were used to analyze the MD simulation results and averaged over the 6 simulations. All images were produced using PyMOL (59). Atom indices for JHFK05 are provided in Table S4.
