# Supplementary figures and images for: Structure-Guided Synthesis of FK506 and FK520 Analogs with Increased Selectivity Exhibit In Vivo Therapeutic Efficacy against Cryptococcus
Source: mBio. 2022 May 23;13(3):e01049-22. doi: 10.1128/mbio.01049-22 (PMC9239059; doi:10.1128/mbio.01049-22)

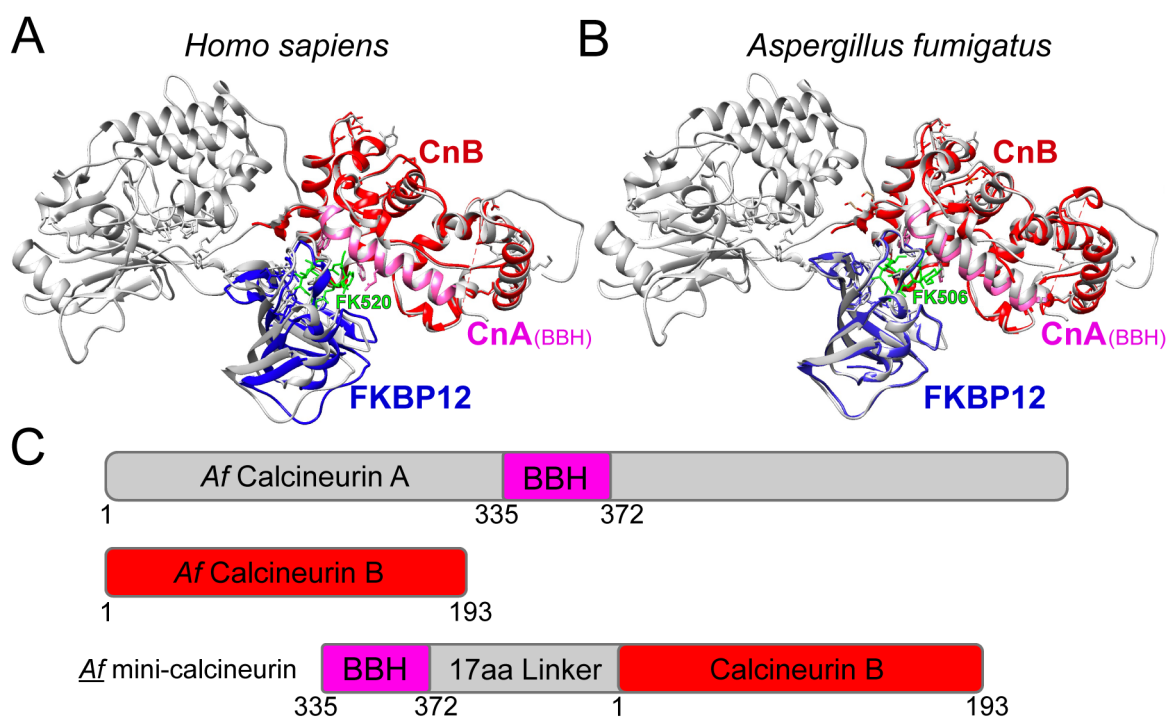

Supplement: FIG S1 [file mbio.01049-22-sf001.pdf]

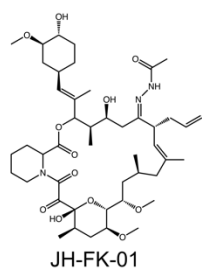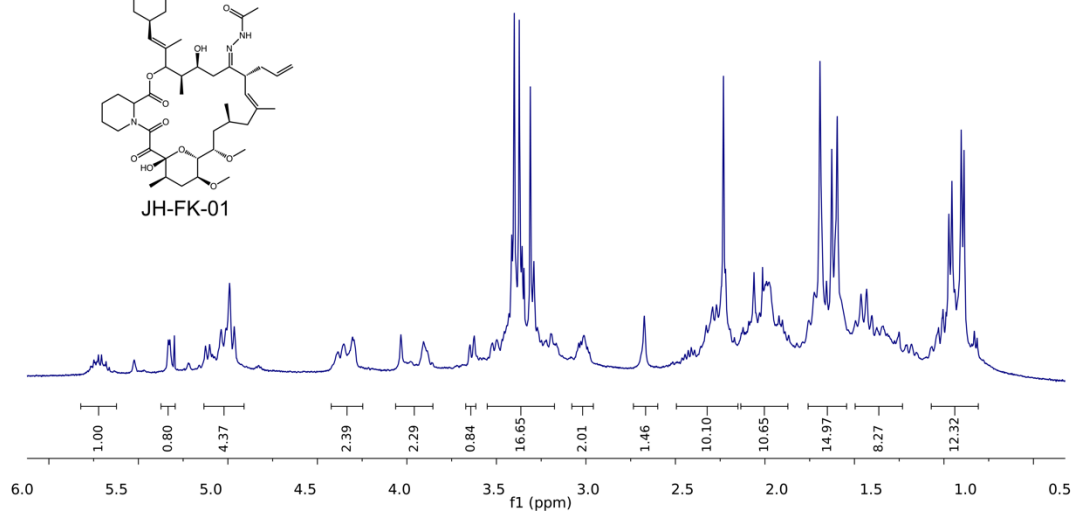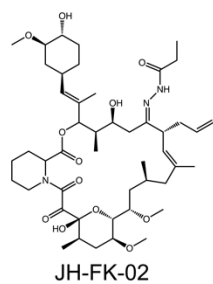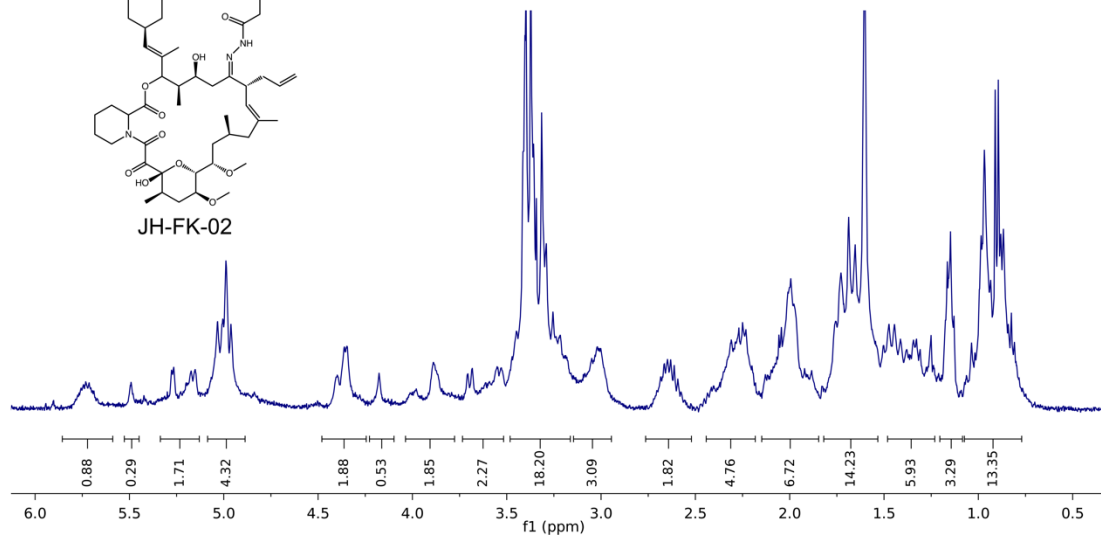

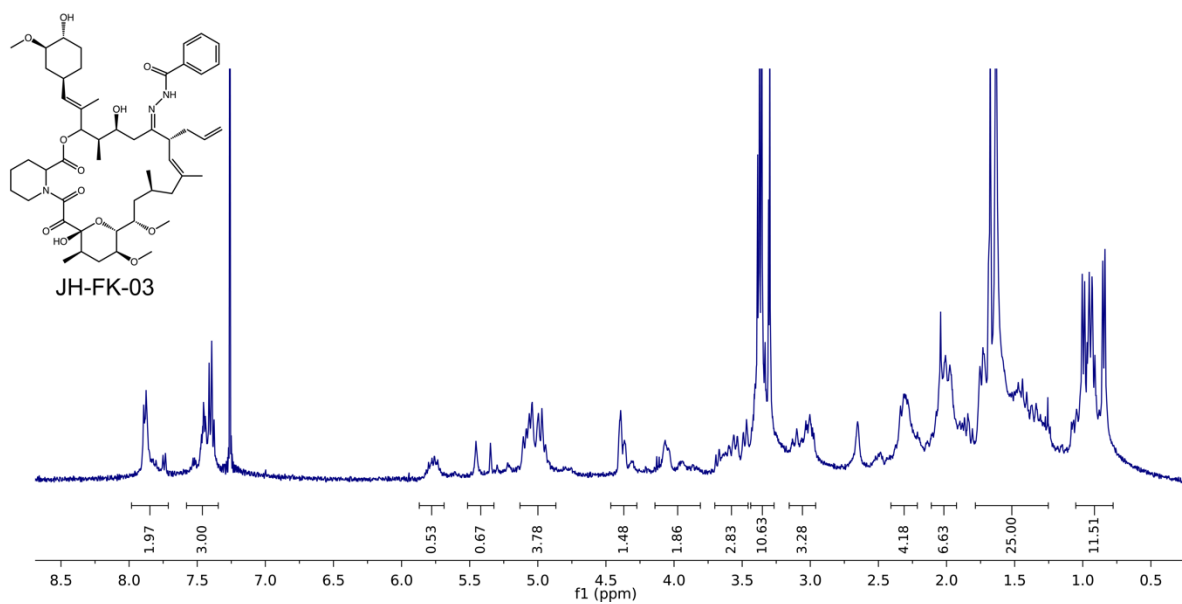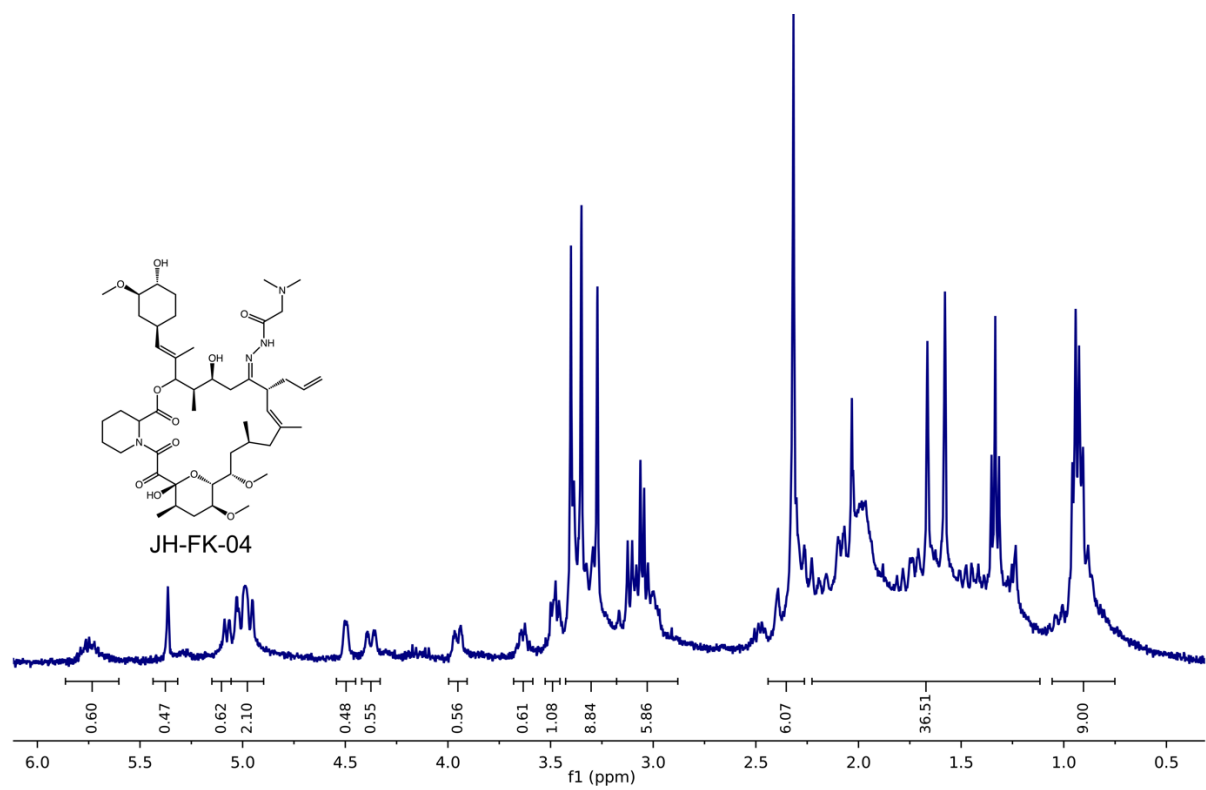

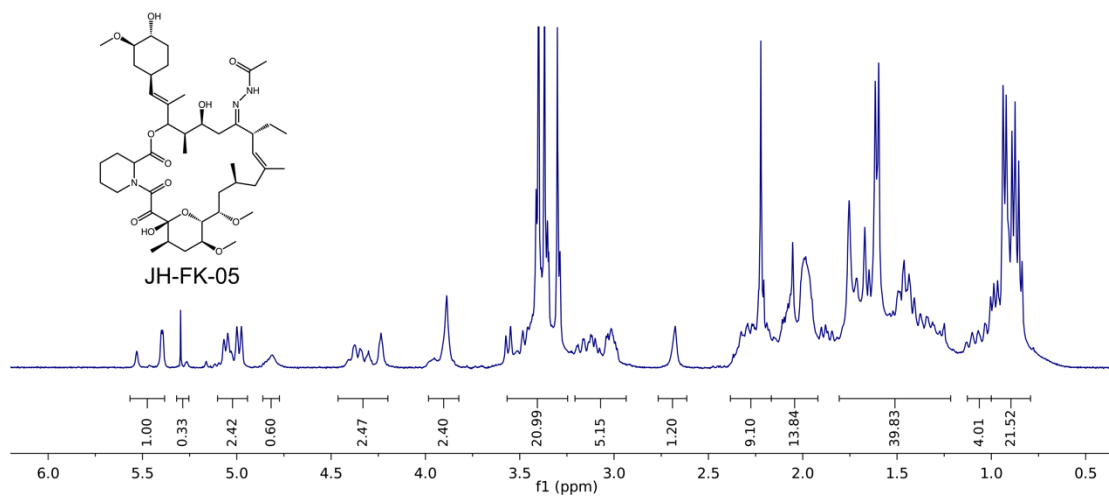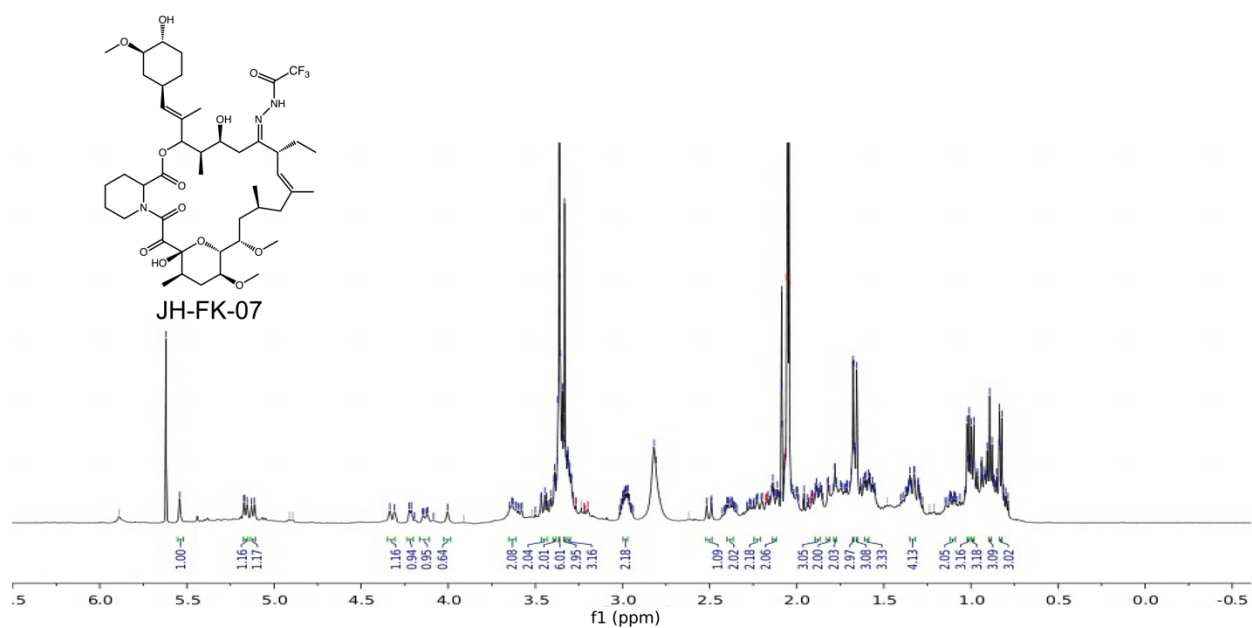

Supplement: FIG S4 [file mbio.01049-22-sf004.pdf]

# Cryptococcus neoformans

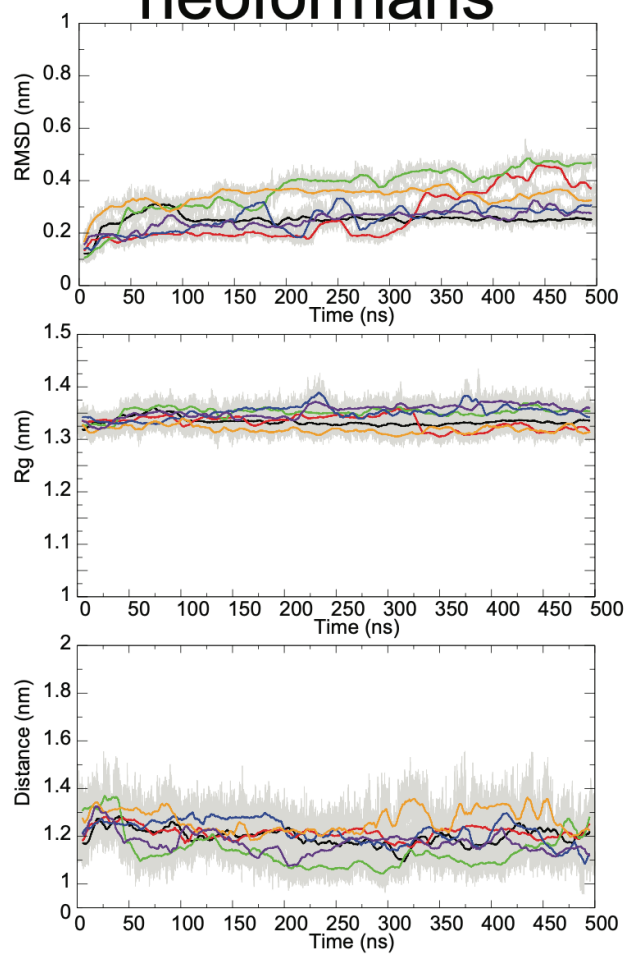

# Human

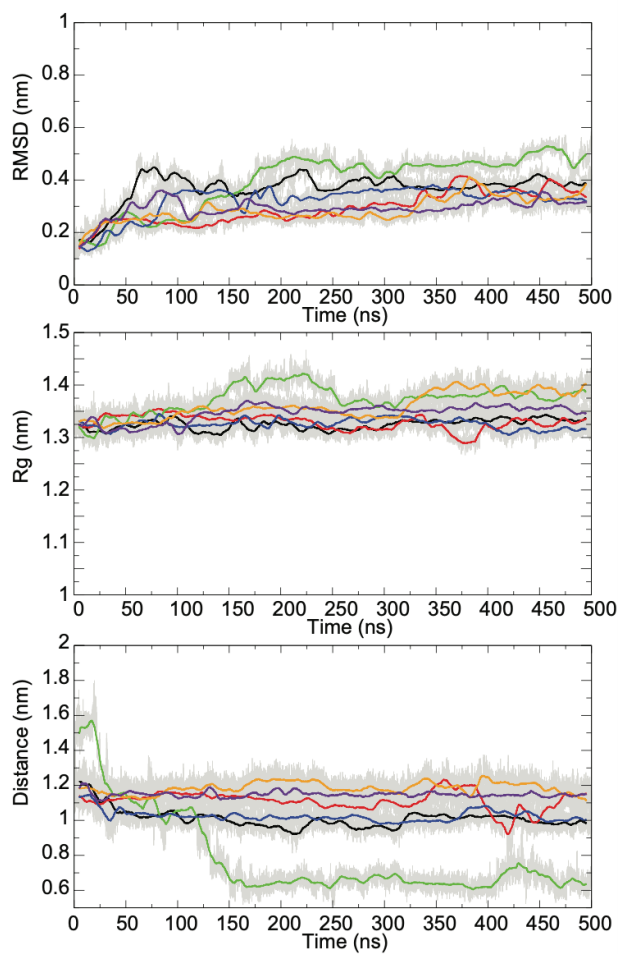

— md1 — md2 — md3 — md4 — md5 — md6

Supplement: FIG S3 [file mbio.01049-22-sf003.pdf]
