# Supplementary material for: Structure-Guided Synthesis of FK506 and FK520 Analogs with Increased Selectivity Exhibit In Vivo Therapeutic Efficacy against Cryptococcus
Source: mBio. 2022 May 23;13(3):e01049-22. doi: 10.1128/mbio.01049-22 (PMC9239059; doi:10.1128/mbio.01049-22)
Supplement: TABLE S1 [file mbio.01049-22-st001.docx]

| Supplementary Table 1. Crystallographic data and refinement statistics | | | |
| --- | --- | --- | --- |
| Data name | *Aspergillus*  *fumigatus*  FKBP12-FK520  PDB 7U0S | *Homo*  *sapiens*  miniCN-FKBP12-FK520  PDB 7U0T | *Aspergillus*  *fumigatus*  miniCN-FKBP12-FK520  PDB 7U0U |
| Data collection |  |  |  |
| Wavelength (Å) | 0.97872 | 0.9786 | 0.9786 |
| Space group | P 2_1_ 2_1_ 2 | C 1 2 1 | I 2 2 2 |
| Cell dimensions |  |  |  |
| a, b, c (Å) | 89.81 91.47 35.53 | 156.1 33.3 64.35 | 75.01 83.7 137.58 |
| α, β, γ (°) | 90.0 90.0 90.0 | 90.0 103.8 90.0 | 90.0 90.0 90.0 |
| Resolution (Å) | 50 - 1.70 (1.74 - 1.70)^a^ | 50 - 2.45 (2.51 - 2.45) | 50 – 1.90 (1.95 – 1.90) |
| No. of unique reflections | 32,881 (2,347) | 11,961 (757) | 34,390 (2,485) |
| R_merge_ (%) | 6.6 (53.0) | 7.1 (37.0) | 4.8 (57.0) |
| I/σ(I) | 13.78 (2.90) | 13.22 (2.64) | 19.94 (2.34) |
| Completeness (%) | 99.5 (99.2) | 97.8 (85.0) | 99.6 (98.5) |
| Redundancy | 4.8 (4.9) | 3.5 (2.7) | 5.9 (4.6) |
| CC½ | 99.8 (88.2) | 99.7 (83.6) | 99.9 (76.5) |
| Refinement |  |  |  |
| Resolution (Å) | 50 – 1.70 | 50 – 2.45 | 50 – 1.90 |
| No. of unique reflections | 32,871 | 11,959 | 34,387 |
| R_work_/R_free_^b^ | 0.168 (0.233)  0.192 (0.269) | 0.203 (0.274)  0.259 (0.338) | 0.169 (0.246)  0.199 (0.261) |
| No. of atoms |  |  |  |
| Protein | 1,845 | 2,274 | 2,415 |
| Ligand | 194 | 64 | 79 |
| Solvent/Water | 227 | 62 | 174 |
| B factors (Å^2^) (overall) |  |  |  |
| Protein | 23.56 | 51.35 | 43.17 |
| Ligand | 29.24 | 31.23 | 32.12 |
| Solvent/Water | 39.25 | 38.81 | 41.99 |
| RMSD |  |  |  |
| Bond lengths (Å) | 0.006 | 0.002 | 0.006 |
| Bond angles (°) | 0.94 | 0.59 | 0.85 |
| Ramachandran favored (%) | 97.84 | 97.94 | 98.34 |
| Ramachandran allowed (%) | 2.16 | 2.06 | 1.66 |
| Ramachandran outliers (%) | 0 | 0 | 0 |
| Rotamer outliers (%) | 1.02 | 1.26 | 1.52 |
| RMSD, root-mean-square deviation. | | | |
| ^a^Highest-resolution shell shown in parentheses. | | | |
| ^b^R_free_ was calculated with 10% of the data | | | |
