# Supplementary material for: Structure-Guided Synthesis of FK506 and FK520 Analogs with Increased Selectivity Exhibit In Vivo Therapeutic Efficacy against Cryptococcus
Source: mBio. 2022 May 23;13(3):e01049-22. doi: 10.1128/mbio.01049-22 (PMC9239059; doi:10.1128/mbio.01049-22)
Supplement: TABLE S2 [file mbio.01049-22-st002.docx]

| **Supplementary Table 2. High-resolution mass spectrometry (HRMS) data for FK506/FK520 analogs** | | | | | |
| --- | --- | --- | --- | --- | --- |
|  | Elemental Composition | Calculated Mass | Observed Mass | Error (ppm) | Ion |
| JH-FK-01 | C_46_H_73_N_3_O_12_ | 860.5267 | 860.5276 | -1 | [M+H]+ |
|  |  | 882.5087 | 882.5091 | -0.5 | [M+Na]+ |
|  |  |  |  |  |  |
| JH-FK-02 | C_47_H_75_N_3_O_12_ | 874.5424 | 874.5429 | -0.6 | [M+H]+ |
|  |  | 896.5243 | 896.5247 | -0.4 | [M+Na]+ |
|  |  |  |  |  |  |
| JH-FK-03 | C_51_H_75_N_3_O_12_ | 922.5424 | 922.5416 | 0.8 | [M+H]+ |
|  |  | 944.5243 | 944.5236 | 0.8 | [M+Na]+ |
|  |  |  |  |  |  |
| JH-FK-04 | C_48_H_78_N_4_O_12_ | 903.5689 | 903.5697 | -0.9 | [M+H]+ |
|  |  | 925.5509 | 925.5507 | 0.1 | [M+Na]+ |
|  |  |  |  |  |  |
| JH-FK-05 | C_45_H_73_N_3_O_12_ | 848.5267 | 848.5273 | -0.7 | [M+H]+ |
|  |  | 870.5087 | 870.5089 | -0.3 | [M+Na]+ |
|  |  |  |  |  |  |
| JH-FK-07 | C_45_H_70_F_3_N_3_O_12_ | 902.0592 | 902.498 | 0.5 | [M+H]+ |
|  |  | 924.4804 | 924.4792 | 1.3 | [M+Na]+ |
