# Supplementary material for: Structure-Guided Synthesis of FK506 and FK520 Analogs with Increased Selectivity Exhibit In Vivo Therapeutic Efficacy against Cryptococcus
Source: mBio. 2022 May 23;13(3):e01049-22. doi: 10.1128/mbio.01049-22 (PMC9239059; doi:10.1128/mbio.01049-22)
Supplement: TABLE S3 [file mbio.01049-22-st003.docx]

| **Supplementary Table 3. Antifungal activity of JH-FK-05 against molds.** | | |
| --- | --- | --- |
| Species | Strain | MIC/MEC (µg/mL) |
| *Aspergillus fumigatus* (Wild-type) | (akuBKu80) | 1 |
| *Aspergillus fumigatus* | (akuBKu80 hFKBP12) | >8 |
| Azole-Resistant Strains  *Aspergillus fumigatus* | F12776 | 2 |
| *Aspergillus fumigatus* | F14946 | 1 |
| *Aspergillus fumigatus* | F16314 | 1 |
| *Aspergillus fumigatus* | F16216 | 1 |
| *Aspergillus fumigatus* | F7075 | 2 |
| Echinocandin-Resistant Strain  *Aspergillus fumigatus* | EMFR-S678P | 2 |
| Other Aspergillus Species  *Aspergillus calidoustus* | WT | >8 |
| *Aspergillus flavus* | WT | 1 |
| *Aspergillus niger* | WT | <1 |
| *Aspergillus terreus* | WT | 1 |
| Other molds  *Fusarium solani* | WT | >8 |
| *Mucor circinelloides* | WT | 1 |
| *Paecilomyces variotti* | WT | >8 |
| *Rhizomucor pusilus* | WT | >8 |
| *Rhizopus oryzae* | WT | 1 |
| *Scedosporium apiospermum* | WT | >8 |
| *Scedosporium prolificans* | WT | >8 |
