# Supplementary material for: Structure-Guided Synthesis of FK506 and FK520 Analogs with Increased Selectivity Exhibit In Vivo Therapeutic Efficacy against Cryptococcus
Source: mBio. 2022 May 23;13(3):e01049-22. doi: 10.1128/mbio.01049-22 (PMC9239059; doi:10.1128/mbio.01049-22)
Supplement: FIG S2 [file mbio.01049-22-sf002.pdf]

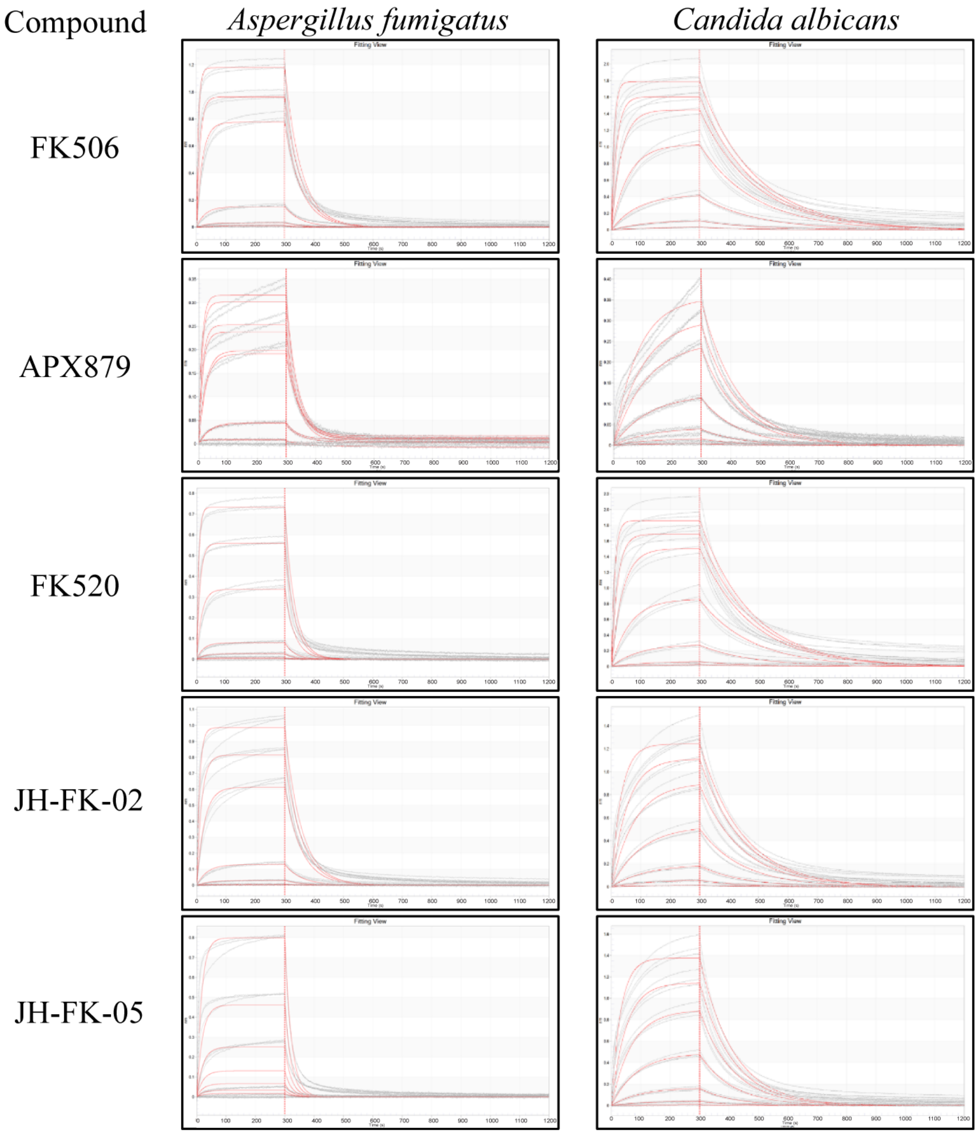

Sample	K_D (M)	K_d (M)	k_{on} ($M^{-1}s^{-1}$)	k_{off} (s^{-1})
***Af* + FK506**	1.5e-6	3.56e-7	5.07e+4	1.81e-2
***Ca* + FK506**	4.7e-7	1.37e-7	3.90e+4	5.32e-3
***Af* + APX879**	1.7e-6	7.00e-7	3.14e+4	2.20e-2
***Ca* + APX879**	1.1e-6	4.76e-6	1.66e+3	7.90e-3
***Af* + FK520**	2.4e-6	6.69e-7	4.52e+4	3.03e-2
***Ca* + FK520**	6.0e-7	1.72e-7	3.30e+4	5.68e-3
***Af* + JH-FK-02**	1.6e-6	5.29e-7	3.38e+4	1.79e-2
***Ca* + JH-FK-02**	8.6e-7	7.34e-7	9.41e+3	6.91e-3
***Af* + JH-FK-05**	7.0e-6	6.73e-6	6.62e+3	4.46e-2
***Ca* + JH-FK-05**	1.1e-6	9.57e-7	9.20e+3	8.80e-3
