# Supplementary material for: Structure-Guided Synthesis of FK506 and FK520 Analogs with Increased Selectivity Exhibit In Vivo Therapeutic Efficacy against Cryptococcus
Source: mBio. 2022 May 23;13(3):e01049-22. doi: 10.1128/mbio.01049-22 (PMC9239059; doi:10.1128/mbio.01049-22)
Supplement: TABLE S4 [file mbio.01049-22-st004.docx]

| **Supplementary Table 4. Atom index for JH-FK-05 MD analysis.** | | | | | | |
| --- | --- | --- | --- | --- | --- | --- |
| Index | Type | Atoms |  | Index | Type | Atoms |
| 1 | C | C01 |  | 31 | CH | C30/H24 |
| 2 | CH | C02/H02 |  | 32 | CH | C31/H25 |
| 3 | CH_2_ | C03/H03/H44 |  | 33 | CH_2_ | C32/H26/H53 |
| 4 | CH_2_ | C04/H04/H45 |  | 34 | CH_2_ | C33/H27/H54 |
| 5 | CH_2_ | C05/H05/H46 |  | 35 | CH_3_ | C34/H28/H55/H66 |
| 6 | CH_2_ | C06/H06/H47 |  | 36 | CH_3_ | C35/H29/H56/H67 |
| 7 | N | N45 |  | 37 | CH_3_ | C36/H30/H57/H68 |
| 8 | C | C07 |  | 38 | CH_2_ | C37/H31/H58 |
| 9 | C | C08 |  | 39 | CH_3_ | C38/H32/H59/H33 |
| 10 | C | C09 |  | 40 | CH_3_ | C40/H34/H60/H69 |
| 11 | CH | C10/H07 |  | 41 | CH_3_ | C41/H35/H61/H70 |
| 12 | CH_2_ | C11/H08/H48 |  | 42 | CH_3_ | C42/H36/H62/H71 |
| 13 | CH | C12/H09 |  | 43 | CH_3_ | C43/H37/H63/H72 |
| 14 | CH | C13/H10 |  | 44 | CH_3_ | C44/H38/H64/H73 |
| 15 | CH | C14/H11 |  | 45 | C | C59 |
| 16 | CH_2_ | C15/H12/H49 |  | 46 | CH_3_ | C60/H01/H43/H65 |
| 17 | CH | C16/H13 |  | 47 | N | N54 |
| 18 | CH_2_ | C17/H14/H50 |  | 48 | NH | N55/H40 |
| 19 | C | C18 |  | 49 | O | O46 |
| 20 | CH | C19/H15 |  | 50 | O | O47 |
| 21 | CH | C20/H16 |  | 51 | O | O48 |
| 22 | C | C21 |  | 52 | O | O49 |
| 23 | CH_2_ | C22/H17/H51 |  | 53 | O | O50 |
| 24 | CH | C23/H18 |  | 54 | OH | O51/H39 |
| 25 | CH | C24/H19 |  | 55 | O | O52 |
| 26 | CH | C25/H20 |  | 56 | O | O53 |
| 27 | C | C26 |  | 57 | OH | O56/H41 |
| 28 | CH | C27/H21 |  | 58 | O | O57 |
| 29 | CH | C28/H22 |  | 59 | OH | O58/H42 |
| 30 | CH_2_ | C29/H23/H52 |  | 60 | O | O61 |
